# The effect of different hemostatic agents following dental extraction in patients under oral antithrombotic therapy: a network meta-analysis

**DOI:** 10.1038/s41598-023-39023-7

**Published:** 2023-08-02

**Authors:** Basel Mahardawi, Sirimanas Jiaranuchart, Sirida Arunjaroensuk, Kevin A. Tompkins, Anupap Somboonsavatdee, Atiphan Pimkhaokham

**Affiliations:** 1 Department of Oral and Maxillofacial Surgery, Faculty of Dentistry, Chulalongkorn University, 34 Henri Dunant Road, Wangmai, Patumwan, Bangkok, 10330 Thailand; 2 Office of Research Affairs, Faculty of Dentistry, Chulalongkorn University, Bangkok, Thailand; 3 Department of Statistics, Chulalongkorn Business School, Chulalongkorn University, Bangkok, Thailand

**Keywords:** Dentistry, Dental conditions, Dental pharmacology, Dental treatments

## Abstract

This network meta-analysis was done to thoroughly evaluate the available literature on the use of different hemostatic agents for dental extraction in patients under oral antithrombotic therapy, aiming to identify the agent with the best/worst performance in bleeding control. Considering that such patients have a higher risk of bleeding, choosing the right hemostatic is essential. Twenty-three randomized clinical trials articles were included after completing the literature search. Cyanoacrylate tissue adhesive showed a reduction in the odds of postoperative bleeding events compared with conventional methods (i.e., gauze/cotton pressure, sutures), with a tendency toward a statistical significance (OR 0.03, P = 0.051). Tranexamic acid was the only agent that demonstrated a significantly lower risk of developing postoperative bleeding events (OR 0.27, P = 0.007). Interestingly, chitosan dental dressing and collagen plug had the shortest time to reach hemostasis. However, they ranked last among all hemostatic agents, regarding bleeding events, revealing higher odds than conventional measures. Therefore, it is concluded that the use of cyanoacrylate tissue adhesive and tranexamic acid gives favorable results in reducing postoperative bleeding events following dental extractions. Although chitosan dental dressing and collagen exhibited a faster time to reach hemostasis, they led to a higher occurrence of bleeding events.

## Introduction

Dental extraction is a common procedure performed in oral and maxillofacial surgery clinics^1^. Numerous potential complications may accompany this procedure, manifesting during the peri- and/or postoperative phase^2,3^. Bleeding is among the most encountered and important complications to consider. Post-extraction bleeding is normally managed with conventional methods (i.e., gauze/cotton pressure, sutures). However, these measures may not be sufficient in situations where a higher degree of bleeding is anticipated, as in patients under oral antithrombotic therapy (OAT). These patients have an increased postoperative risk of hemorrhage, in addition to the possibility for a prolonged bleeding time once this event occurs^4,5^.

To reduce the risk of hemorrhage with patients taking antithrombotic medications, it was suggested earlier to stop or modify their drug regimen for a certain period before undergoing dental extractions. Nevertheless, the elevated possibility of developing thromboembolism events^5^, along with other reports demonstrating the ability to properly control postoperative bleeding with these individuals utilizing hemostatic measures^6,7^, led this protocol to become suboptimal. Several studies have investigated the outcome of dental extraction without stopping or modifying the antithrombotic treatment. Their results revealed that, when obtaining sufficient hemostasis upon the completion of this procedure, tooth extraction could be done safely without any adjustment to the patients’ antithrombotic therapy^8–11^. Consequently, this step is not justified and has become less popular.

Although tooth extraction has been shown to be safe in patients with higher bleeding risk, postoperative bleeding events have still been reported^12^. Therefore, finding the best supplementary measures for achieving more effective hemostasis has become vital.

Hemostatic agents have been introduced as a method to secure better hemostasis. Several agents were presented and compared, whether with conventional measures or with other agents^13^. Yet, the best agent among all the available options is still undetermined. Previous systematic reviews have been presented on this topic. However, these studies either lacked quantitative synthesis^14^, or focused on the effect of hemostatic agents in general, compared with the conventional measures^15^. Thus, understanding which agent or agents provide better bleeding control, as well as the ones with less desirable outcomes, would be of a high value.

A recent method of conducting meta-analyses has been proposed, the network meta-analysis. This type of analysis allows for making indirect comparisons between different materials used for the same purpose (hemostatic agents in this case), based on the results of the available direct comparisons, i.e., documented clinical trials^16^. This offers more insight into the hemostatic agents currently used and enriches the available information on their potential. A previous network meta-analysis has been performed on this topic^17^. Nevertheless, the authors combined all types of oral surgical procedures and did not focus on dental extraction in particular. Furthermore, only the agents with the best potential in bleeding control were mentioned and discussed, without emphasis on the ones that may lead to a higher occurrence of postoperative bleeding events. Out of these points, the goal of this network meta-analysis was to thoroughly evaluate the literature on the use of different hemostatic agents for dental extraction, attempting to provide evidence on the agents with the best or worst performance in bleeding control in patients under OAT.

## Results

### Search outcomes

**Figure 1:**
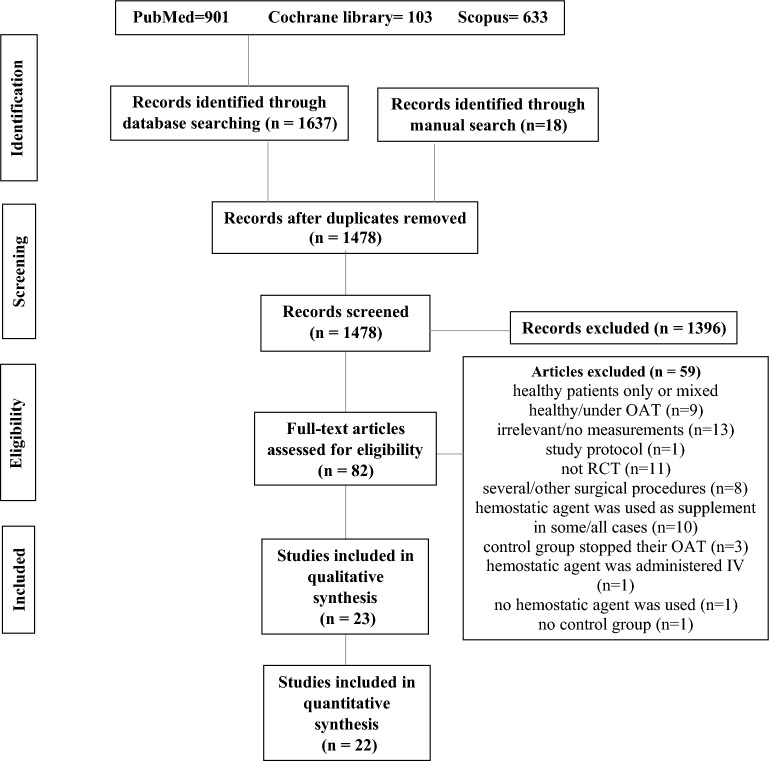
PRISMA flowchart of the search process and outcomes.

There were 1655 identified studies. After removing the duplicates, and screening the titles and abstracts, 82 articles were qualified for the full-text assessment stage. Of these, 59 studies were excluded (Supplementary Table 1). As a result, the search process ended with including 23 articles in this study, 22 of which were eligible for meta-analysis. Figure 1 is the PRIMSA flowchart of the search process and outcomes.

### Study characteristics

Thirteen studies allocated different patients for each group^7,18–29^, while nine had a split-mouth design^6,30–37^. In addition, one investigation had some of the patients being allocated to more than one group (split-mouth for some cases)^38^. Three studies recruited patients on antithrombotic therapy in general without specifying^25,30,33^, while 14 publications stated that patients were on anticoagulants, and 6 investigations enrolled patients on antiplatelet drugs^6,22,31,32,34,35^.

The type of extraction was simple in 19 studies, surgical in one^26^, both simple and surgical two^27,28^, while this was not specified in one publication^29^.

The hemostatic agents used were Chitosan dental dressing^19,30,31,33–37^, Oxidized regenerated cellulose^6,27,32^, Tranexamic acid^20,21,24,28,29,38^, Fibrin sponge or adhesive^18,27,38^, Collagen plug^18,34,37^, Bismuth subgallate^18^, Gelatin sponge^19,22,23,26^, Feracrylum^20^, Epsilon aminocaproic acid^7,29^, Ankaferd blood stopper^25^, and Cyanoacrylate tissue adhesive^26^.

Sixteen studies had a group that received conventional measures, which were the application of gauze pressure^20,24,25,31,36,38^, cotton pressure^30^, or sutures^6,7,21–23,28,32^. Two studies applied gauze pressure, however, used sutures when the case necessitated^33,35^.

The time to reach hemostasis was reported in eleven studies. Eight of them compared a hemostatic agent with conventional measures^21,24,25,30,31,33,35,36^, while 3 compared different agents^26,34,37^. When the agents were compared with conventional measures, six studies stated that hemostatics led to significantly less time to reach hemostasis, whereas 2 studies did not report information about statistical significance^25,31^.

Sixteen studies reported information related to postoperative bleeding events. Among these, five compared different agents^18,19,26,27,29^, while the rest compared one or more hemostatic agents to conventional methods^6,7,20–23,25,28,32,35,38^. The postoperative period to record bleeding events lasted up to 10 days^26^. There was no significant difference between the hemostatic agents and the conventional methods in all studies. In contrast, when evaluating different agents, cyanoacrylate tissue adhesive resulted in significantly fewer bleeding events, as opposed to using a gelatin sponge^26^, while the latter demonstrated a statistical significance compared with chitosan^19^. Moreover, both fibrin and bismuth subgallate were significantly better than collagen^18^. Table 1 summarizes the characteristics of the included studies.

**Table 1:** Characteristics and outcomes of the included studies in this systematic review.

Author	Study design	Patient condition	Type of extraction	Hemostatic agent	Control	Number of patients Number of extractions (hemostatic)	Number of patients Number of extractions (control)	Time to achieve hemostasis (s) (Hemostatic/control)	Bleeding	
									Bleeding events (hemostatic/control)	Postoperative period of occurrence
Radhakrishna (2023)	RCT Split-mouth	On antithrombotic therapy	Simple	Chitosan dental dressing	Cotton pressure pack	54	54	96/797		
						54	54			
Brancaccio (2021)	RCT Split-mouth	On antiplatelet drugs	Simple	Oxidized regenerated cellulose	Suture	102	102	–	12/20	30 min
						102	102			
Ockerman (2021)	RCT	Anticoagulated	Simple/surgical	Tranexamic acid	Suture*	106	112	–	4/10	7 days
						N/A	N/A			
Redwan (2020)	RCT Split-mouth	On antiplatelet drugs	Simple	Chitosan dental dressing	Gauze pressure	40	40	67.8/573	–	–
						40	40			
Puia (2020)	RCT	Anticoagulated	Simple	Fibrin tissue adhesive	–	80	–		1	3 days
						91				
				Collagen plug		80			10	
						85				
				Bismuth subgallate		80			0	
						91				
Giudice (2019)	RCT Split-mouth	On antiplatelet drugs	Simple	Oxidized regenerated cellulose	Suture	40	40	–	5/8	30 min
						40	40			
Seethamsetty (2019)	RCT Split-mouth	On antithrombotic therapy	Simple	Chitosan dental dressing	Gauze pressure + suture if required	40	40	37.8/546		
						40	40			
Ragab (2019)	RCT	Anticoagulated	Simple	Gelatin sponge	–	20	–	–	0	5 min
						20				
				Chitosan dental dressing		20			4	
						20				
Rai (2019)	RCT	Anticoagulated	Simple	Feracrylum	Gauze pressure	20	20	–	1/3	Day of extraction
						20	20			
				Tranexamic acid		20		–	0/3	
						20				
Queiroz (2018)	RCT	Anticoagulated	Simple	Tranexamic acid	Suture	17	20	354/714	0/3	1 day
						17	20			
da Silva (2018)	RCT	Anticoagulated	Simple	Epsilon aminocaproic acid (EACA)	Suture	N/A (total number of patients 52)	N/A (total number of patients 52)	–	11/12	7 days
						70	70			
Pippi (2017)	RCT Split-mouth	On antiplatelet drugs	Simple	Chitosan dental dressing	–	20	–	455.40	–	–
						20				
				Collagen plug		20		282.15		
						20				
Sharma (2017)	RCT Split-mouth	On antiplatelet drugs	Simple	Chitosan dental dressing	Gauze pressure + suture if required	40	40	67.8/840.6	0/1	6 h
						40	40			
Muralidharan (2017)	RCT	On antiplatelet drugs	Simple	Gelatin sponge	Suture	44	44	–	0/2	7 days
						44	44			
Kumar (2016)	RCT Split-mouth	Anticoagulated	Simple	Chitosan dental dressing	Gauze pressure	30	30	89.4/243.6	–	–
						30	30			
Pippi (2015)	RCT Split-mouth	Anticoagulated	Simple	Chitosan dental dressing	–	20	–	279.60	–	–
						20				
				Collagen plug		20		244.90		
						20				
Soares (2015)	RCT (Split-mouth for some cases)	Anticoagulated	Simple	Tranexamic acid	Gauze pressure	12	13	–	1/1	7 days
						28	28			
				Fibrin sponge		13		–	2/1	
						28				
Bajkin (2014)	RCT	Anticoagulated	Simple	Gelatin sponge	Suture	30	30	–	2/1	Several hours
						40	42			
Ripolles-de Ramon (2014)	RCT	Anticoagulated	Simple	Tranexamic acid	Gauze pressure	8	8	Median (evaluated at 15-min intervals) 30/90		
						N/A	N/A			
Cakarer (2013)	RCT	On antithrombotic therapy	Simple	Ankaferd blood stopper	Gauze pressure	15	10	56.2/204	0/1	1 day
						20	12			
Al-Belasy (2003)	RCT	Anticoagulated	Surgical	Cyanoacrylate tissue adhesive	–	15 Mean = 6	–	Immediately	0	10 days
				Gelatin sponge		15 Mean = 6.3		10–20 min	5	
Halfpenny (2001)	RCT	Anticoagulated	Simple/surgical	Fibrin tissue adhesive	–	20 Mean 2/patient	–	–	2	1 week
				Oxidized regenerated cellulose		26 Mean 1.5/patient			1	
Souto (1996)	RCT	Anticoagulated	–	Tranexamic acid	–	12	–	–	2	2 days
				Epsilon aminocaproic acid (EACA)		13			4	

*RCT* randomized clinical trial.

*The control group received a placebo that does not have any hemostatic effect.

### Risk of bias

Upon implementing the RoB2 tool, eight studies showed a low risk of bias^7,18,21,28,31,32,34,37^. These studies had a clear randomization process, well-described intervention with no potential deviation from it, and no/little loss to follow-up, in addition to an appropriate and clear measuring and reporting of the outcome. Moreover, fourteen studies had some concerns^6,19,20,22,23,25–27,29,30,33,35,36,38^, mostly due to an unclear randomization process, while one investigation was judged to be of a high risk of bias^24^. The main reason was the questionable method of measuring/reporting the outcome (Supplementary Fig. 1).

### Data synthesis and network meta-analysis

#### Time to reach hemostasis

**Figure 2:**
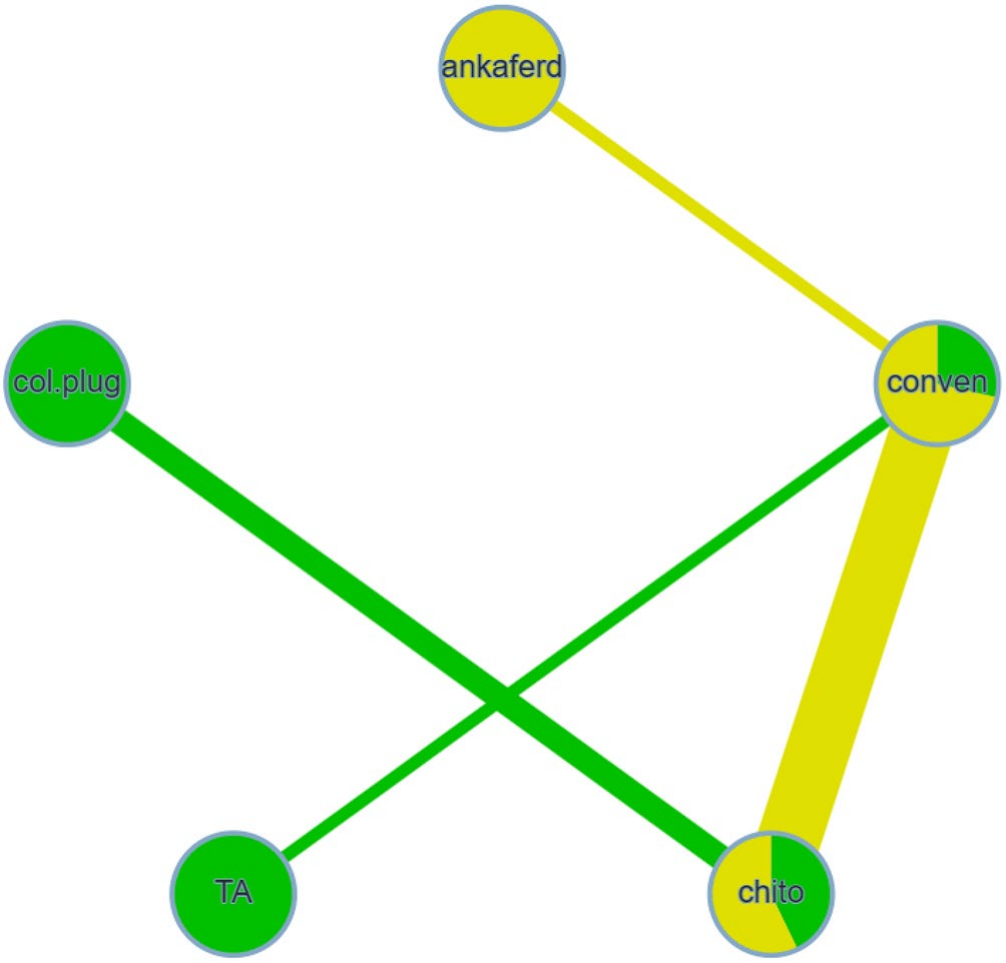
Network meta-analysis geometry of the comparisons available on the time to reach hemostasis. Each node represents an intervention. The line between 2 nodes represents a direct comparison that has been conducted in a clinical trial. The thicker the line, the more direct comparisons available. The color of the node is based on the number and risk of bias of the studies that included the relevant agent, where green and yellow represent “low risk” and “some concerns”, respectively. The color of the line is based on the risk of bias in the majority of the studies that included the relevant comparison. *Ankaferd* ankaferd blood stopper, *col.plug* collagen plug, *conven* conventional methods, *TA* tranexamic acid, *chtio* chitosan dental dressing.

**Figure 3:**
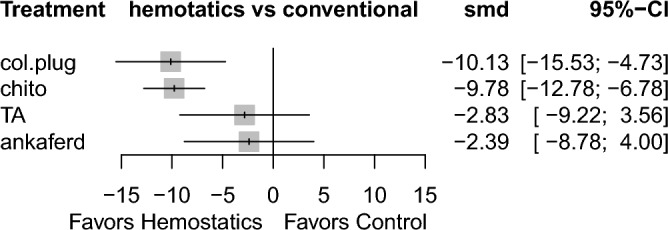
Forest plot to show the ranking of the available interventions with regards to the time to reach hemostasis.

Nine studies were included in this network meta-analysis^21,25,30,31,33–37^, having 5 methods used to achieve hemostasis. The type of extraction was simple in all of these studies. Figure 2 illustrates the comparisons performed in the included investigations. Substantial heterogeneity and inconsistency between the studies were found (I^2^ = 98.3%, Q = 288.97, P < 0.0001). The results of this meta-analysis revealed that chitosan dental dressing, as well as collagen plug led to significantly faster hemostasis, compared with the conventional methods (SMD =  − 9.78, 95% CI − 12.78 to − 6.78, and SMD =  − 10.13, 95% CI − 15.53 to − 4.73, respectively) (Table 2, Supplementary Table 2). This was based on direct and indirect evidence, respectively. The league table also indicated that ankaferd blood stopper led to a significantly longer time to reach hemostasis, compared with chitosan (SMD = 7.39, 95% CI 0.33 to 14.45), based on indirect evidence (Table 2). Treatment ranking revealed that chitosan and collagen are likely to perform best in reducing the time to reach hemostasis (Fig. 3).

**Table 2:**
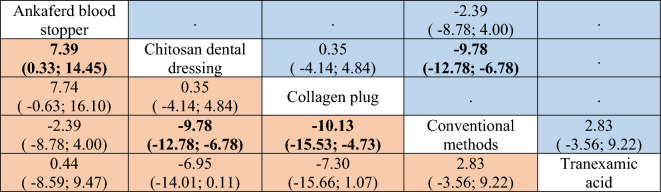
League table of the network meta-analysis on the time to reach hemostasis.

Results highlighted in blue represent direct comparisons, while the ones in orange are based on indirect evidence. Each estimate in the cell is the vertex of a 90-degree angle, where the ends of the two arms are the compared treatments (from left to right). Results in bold indicate statistical significance.

#### Bleeding events

**Figure 4:**
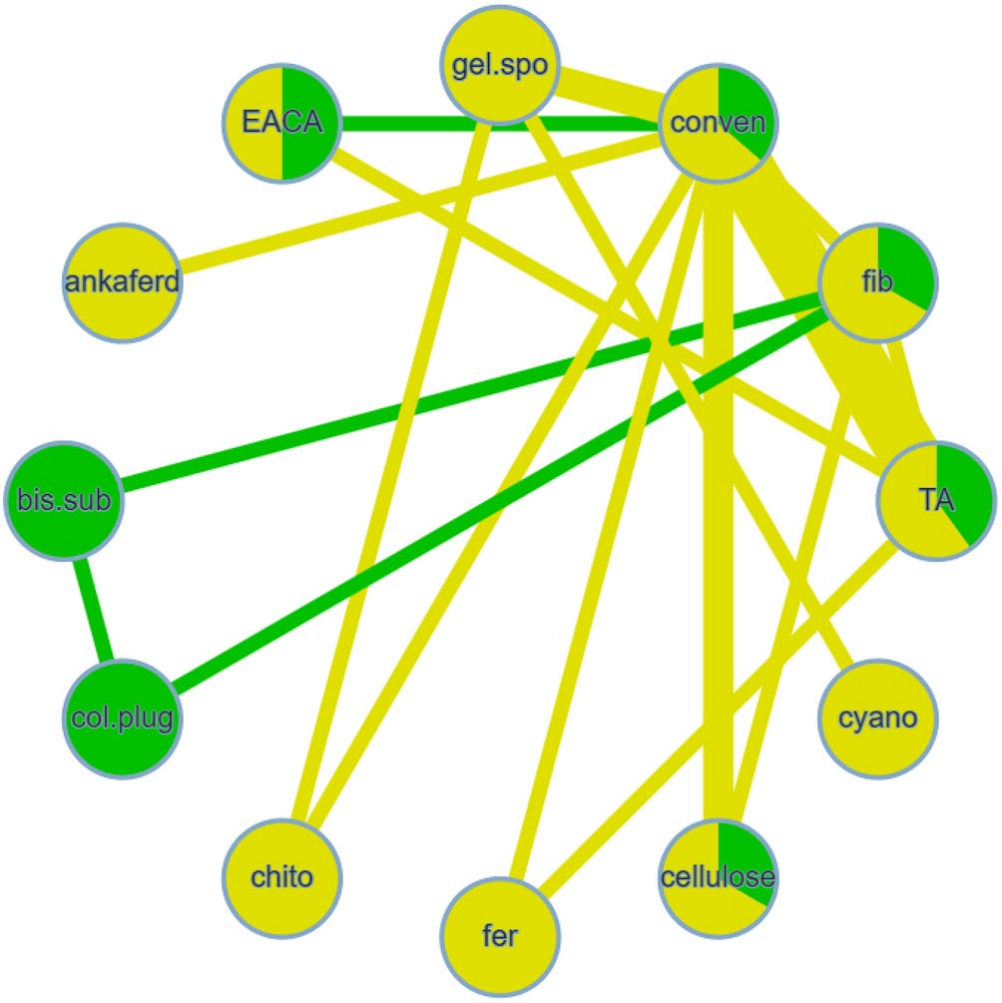
Network meta-analysis geometry of the comparisons available on bleeding events. *Ankaferd* ankaferd blood stopper, *col.plug* collagen plug, *conven* conventional methods, *TA* tranexamic acid, *chtio* chitosan dental dressing, *EACA* Epsilon aminocaproic acid, *bis.sub* Bismuth subgallate, *fib* fibrin *gel.spo* gelatin sponge, *cyano* cyanoacrylate tissue adhesive, *cellulose* Oxidized regenerated cellulose, *fer* Feracrylum.

**Figure 5:**
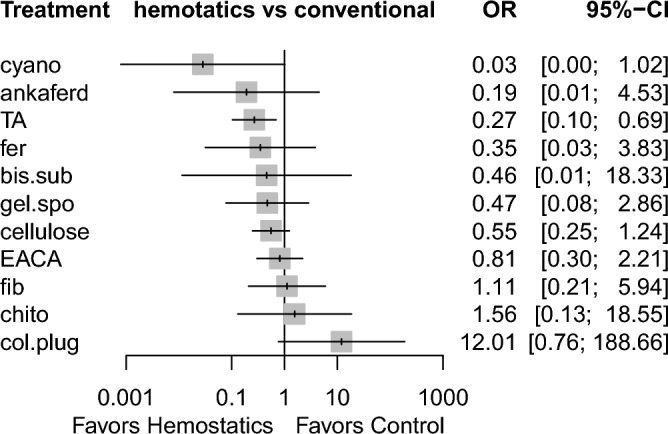
Forest plot to show the ranking of the available interventions with regards to bleeding events.

Sixteen studies reported information related to this outcome^6,7,18–23,25–29,32,35,38^, based on 12 different methods used. Figure 4 demonstrates the comparisons performed among these investigations. Low heterogeneity (I^2^ = 12.5%) and inconsistency (Q = 3.10; P-value = 0.69) were noted. Tranexamic acid was the only agent that showed a significant difference compared with conventional methods (OR 0.27, 95% CI 0.10 to 0.69). In addition, cyanoacrylate tissue adhesive showed a tendency towards statistical significance (OR 0.03, 95% CI 0.0008 to 1.02, P = 0.051) (Table 3, Supplementary Table 3), based on indirect evidence. Comparing different agents, bismuth subgallate and fibrin showed significantly lower odds of postoperative bleeding events, as opposed to collagen plug. This was based on direct evidence. As for the indirect evidence, chitosan dental dressing demonstrated significantly higher odds, compared with cyanoacrylate and tranexamic acid. Collagen plug also showed significantly higher odds than cyanoacrylate and tranexamic acid (Table 3). Treatment ranking revealed that cyanoacrylate tissue adhesive is likely to show the best results. However, tranexamic acid had the narrowest confidence interval (with statistical significance). Chitosan dental dressing and collagen plug ranked last among all hemostatic agents (Fig. 5).

**Table 3:**
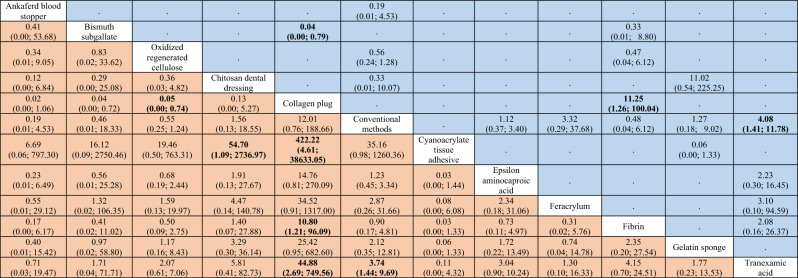
League table of the network meta-analysis of bleeding events in antithrombotic patients.

Results highlighted in blue represent direct comparisons, while the ones in orange are based on indirect evidence.

#### Bleeding events in anticoagulated patients

**Figure 6:**
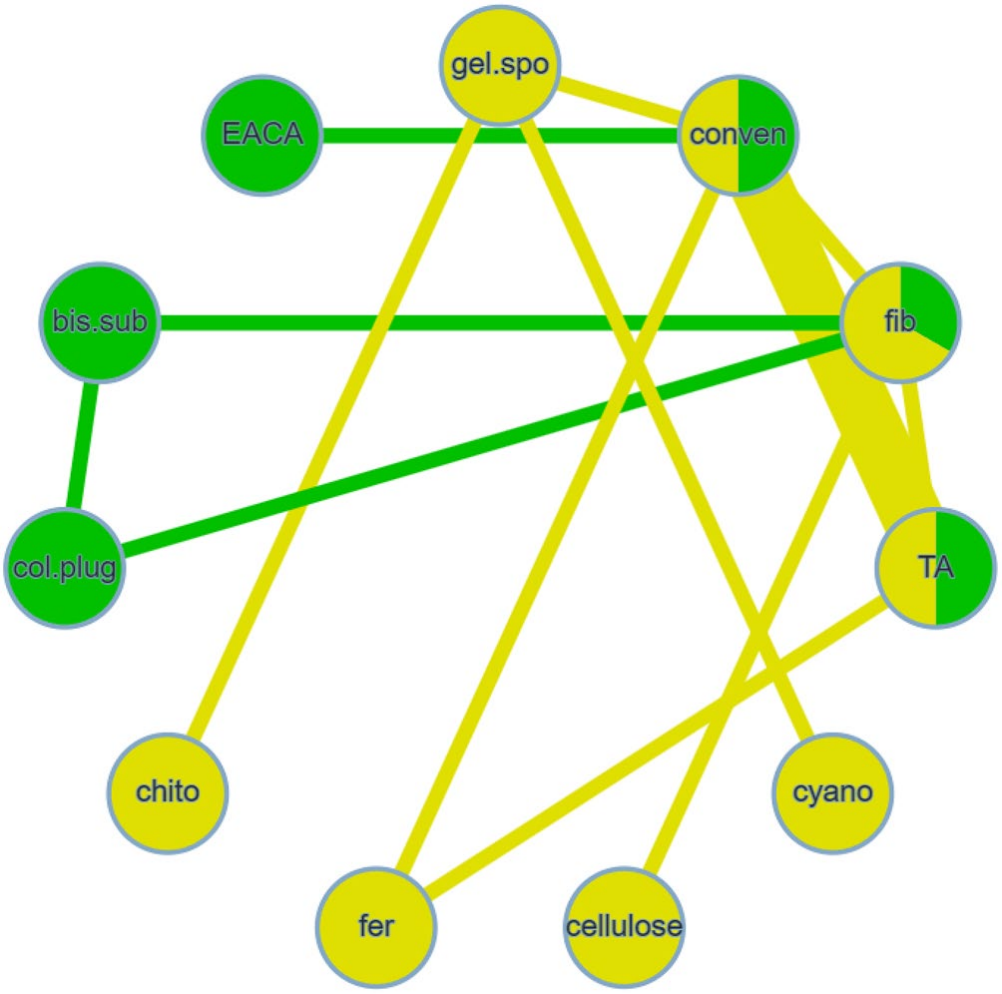
Network meta-analysis geometry of the comparisons available on bleeding events in anticoagulated patients.

**Figure 7:**
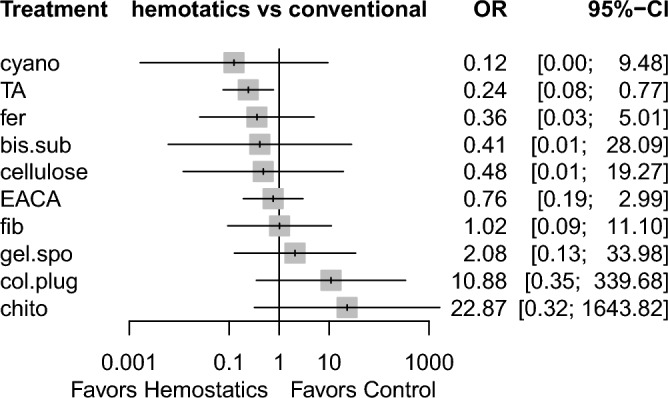
Forest plot to show the ranking of the available interventions with regards to bleeding events in anticoagulated patients.

Eleven investigations were analyzed, including 11 different treatments^7,18–21,23,26–29,38^. Figure 6 shows the methods compared in the analyzed studies. Moderate heterogeneity (I^2^ = 32.3%), with no significant inconsistency (Q = 1.33; P-value = 0.7217) was seen. Similarly, tranexamic acid only showed statistical significance, compared with conventional methods (OR 0.24, 95% CI 0.08 to 0.77) (Table 4, Supplementary Table 4). Concerning different agents, bismuth subgallate demonstrated significantly lower odds of postoperative hemorrhage events than collagen plug. Chitosan dental dressing indicated significantly higher odds, compared with cyanoacrylate and tranexamic acid. Collagen plug showed significantly higher odds than tranexamic acid. These comparisons were based on indirect evidence (Table 4). Treatment ranking revealed that cyanoacrylate tissue adhesive had the lowest odds of postoperative bleeding. However, tranexamic acid obtained the narrowest confidence interval. Chitosan dental dressing and collagen plug also ranked the worst among all hemostatic agents (Fig. 7).

**Table 4:**
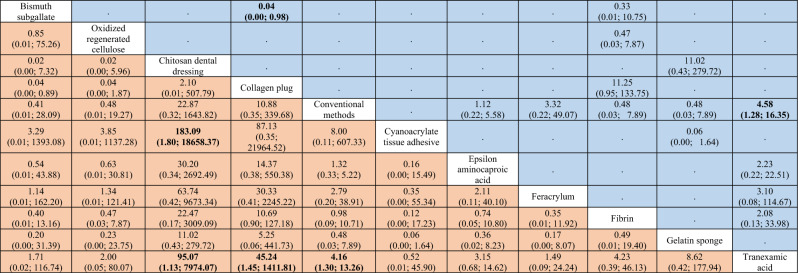
League table of the network meta-analysis of bleeding events in anticoagulated patients.

Results highlighted in blue represent direct comparisons, while the ones in orange are based on indirect evidence.

#### Bleeding events after simple tooth extraction

**Figure 8:**
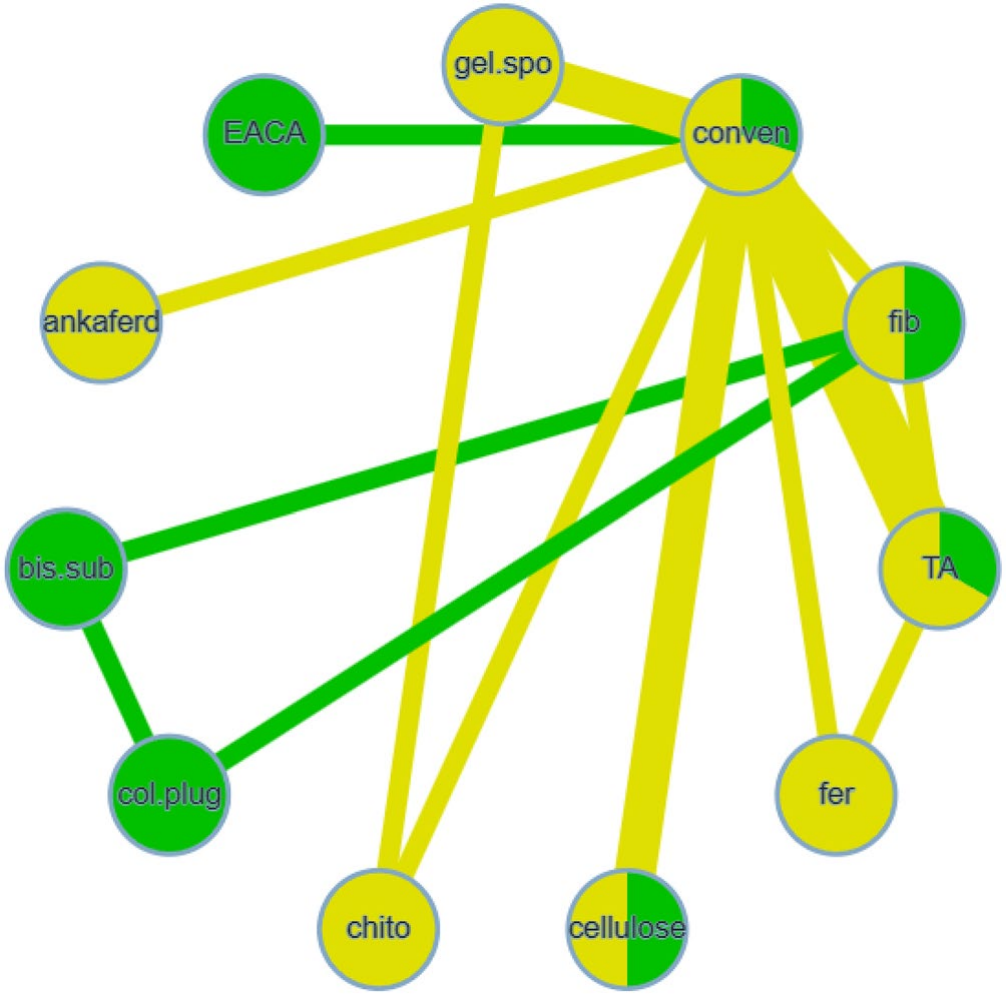
Network meta-analysis geometry of the comparisons available on bleeding events after simple (i.e., non-surgical) tooth extraction.

**Figure 9:**
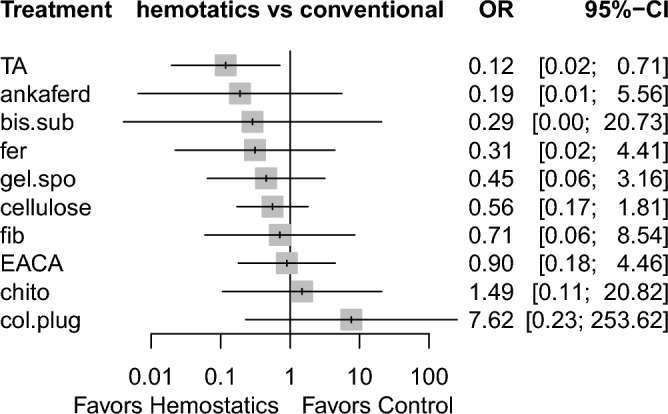
Forest plot to show the ranking of the available interventions with regards to bleeding events after simple tooth extraction.

As the majority of the studies recruited patients undergoing simple dental extractions, a subgroup analysis was performed, in order to further validate the evidence provided from the previous analyses, including more homogenous investigations. Twelve studies, with 11 treatments, were analyzed^6,7,18–23,25,32,35,38^. Figure 8 illustrates the comparisons performed among these clinical trials. The level of heterogeneity was moderate (I^2^ = 34.3%) and no significant inconsistency was identified (Q = 6.13, P-value = 0.11). Tranexamic acid also showed a significant effect in reducing bleeding events, compared with the conventional measures (OR 0.12, 95% CI 0.02 to 0.71) (Table 5, Supplementary Table 5). When comparing different hemostatic agents, bismuth subgallate and fibrin demonstrated significantly lower odds of postoperative hemorrhage than collagen plug based on direct evidence. The same was noted when comparing tranexamic acid and collagen plug, as indicated by the indirect evidence (Table 5). Treatment ranking revealed that tranexamic acid is likely to perform best among all hemostatic agents, while chitosan and collagen ranked last, as described in the previous analyses (Fig. 9). The meta-analysis done with 7 studies^7,18–21,23,38^ including anticoagulated patients who underwent simple tooth extraction also showed a similar trend (Supplementary Figs. 2, 3, Supplementary Tables 6 and 7).

**Table 5:**
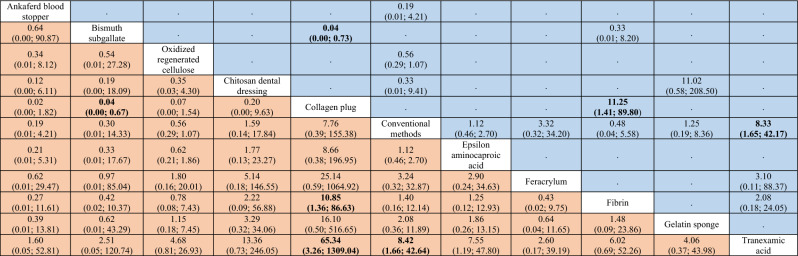
League table of the network meta-analysis of bleeding events after simple (i.e., non-surgical) tooth extractions in antithrombotic patients.

Results highlighted in blue represent direct comparisons, while the ones in orange are based on indirect evidence.

### Confidence in the evidence of network meta-analysis outcomes

The certainty of evidence for all outcomes is presented in Supplementary Tables 8–12. Chitosan and collagen plug were the agents that demonstrated a significant difference compared with conventional measures, regarding the time to achieve hemostasis. However, the compared studies had potential bias, heterogeneity, and incoherence, leading to a low to very low level of evidence. The level of evidence for all other comparisons under this outcome was also concluded to be either low or very low.

As for bleeding events in antithrombotic patients in general or anticoagulated patients in particular, the evidence on the efficacy of tranexamic acid was downgraded by one level to become moderate. The reason was the possible heterogeneity between the studies.

With respect to the comparison between agents that demonstrated statistical significance, these were also at a moderate to low level of evidence.

The indirect evidence on the superiority of tranexamic acid compared with collagen plug was high when analyzing all studies, however, it was downgraded to moderate when the analysis was done on anticoagulated patients specifically, due to the imprecision of the result. In addition, the evidence was further downgraded to be low when including studies with simple extraction only, because of the potential bias in the analyzed investigations as well as the imprecision of the overall outcome.

## Discussion

As presented in this study, numerous hemostatic agents have been used and compared with conventional methods for bleeding control after dental extractions. Choosing one agent over the other or concluding which method is more effective was not possible, especially since only some agents have been compared and the majority have been used in different studies. Consequently, this network meta-analysis was conducted, aiming to provide a comprehensive evaluation of the available hemostatic agents and give an idea on the agents that perform best in bleeding control, as well as the ones with unfavorable results.

Among all hemostatic agents compared in this network meta-analysis, cyanoacrylate tissue adhesive ranked as the one with the least odds of postoperative bleeding in patients on antithrombotic therapy. This agent resulted in a notable decrease in the risk of postoperative bleeding events (OR 0.03). Cyanoacrylate tissue adhesive has been presented as an alternative to the conventional methods of hemostasis, and applied in several surgical procedures^26,39,40^, including tooth extraction^26^. It has gained increased interest, due to its efficacy in bleeding control^26^, adherence to soft and hard tissues^41,42^, antimicrobial potential, as well as its relatively easy application, which aids in effective tissue handling and results in overall shorter operation time^41,43^. Cyanoacrylate tissue adhesive has also been compared with sutures for mandibular third molar surgery in several studies on healthy patients, and led to a lower occurrence of postoperative bleeding events^43,44^, as well as a reduction in the degree of pain^44,45^. It is worth mentioning that although no significant difference was noted in this study, the high end of the 95% CI was 1.02, which indicates a tendency towards statistical significance (P = 0.051). Therefore, all previous studies, accompanied by the evidence presented in this network meta-analysis, indicate the efficacy of this hemostatic agent in reducing the chance of postoperative bleeding when applied for closing the site after surgical extraction.

The only agent that showed statistical significance in reducing postoperative hemorrhage events was tranexamic acid. It demonstrated an approximate 70% decrease in the likelihood of developing postoperative bleeding events (OR 0.27), with a narrow confidence interval, revealing the high possibility of this outcome to be clinically meaningful, rather than just hypothetical. This hemostatic agent has been used for a long time in bleeding control in oral surgery, benefiting from its low systemic resorption when applied topically^46,47^. A previous meta-analysis concluded that tranexamic acid was effective in reducing postoperative bleeding events, compared with other agents or a placebo^48^. Another systematic review pointed out that this agent seems to provide better results in bleeding control, however, conclusive evidence is still unavailable^14^. The outcome of the present meta-analysis confirms what is stated in the literature, showing the superiority of tranexamic acid in reducing the occurrence of postoperative bleeding events. Therefore, it should be among the first options to consider when performing dental extractions in patients on OAT.

The efficacy of tranexamic acid and cyanoacrylate, concluded from this meta-analysis, is in line with an earlier report on different oral surgical procedures^17^. However, the most notable finding was probably the one regarding chitosan dental dressing and collagen plug. As shown in the analysis of time to reach hemostasis, these agents ranked the highest, revealing a high statistical significance (SMD =  − 9.78, P < 0.0001 and SMD =  − 10.13, P = 0.0002, respectively), i.e., a major reduction in the time to achieve hemostasis. Conversely, they ranked last in controlling post-extraction bleeding events, when the analysis was done on antithrombotic patients in general or anticoagulated patients only. Their efficacy was even worse than the conventional methods of hemostasis. This could also support the ‘low/very low’ level of evidence on their effectiveness in reducing the time to reach hemostasis. Chitosan in general promotes wound healing, enhances the production of platelet-derived growth factors^49^, and has been shown to possess antimicrobial characteristics^50^, which also play a role in the overall healing of extraction sites. In contrast, some chitosan dressings have poor mechanical properties^51,52^, which could lead to later dislodgement from the socket, and consequently, postoperative bleeding. Moreover, collagen has very good biocompatibility and cell adhesion. This porous or fibrous sponge is non-toxic and can be used in various clinical scenarios where hemostasis is needed^53^. Similar to chitosan, collagen possesses low mechanical strength and an unpredictable biodegradation rate^53–55^. Although prompt hemostasis is secured with the use of this agent, these disadvantages may contribute to the bleeding events present at a later stage. Therefore, considering all the previous points, and based on the results of this meta-analysis, choosing other hemostatic agents, such as tranexamic acid is preferable over the use of collagen or chitosan dressing. It is also noteworthy that when the analysis was done on patients taking anticoagulants, gelatin sponge performed worse than conventional measures. A possible cause is that sponges can expand multiple times in size compared with their baseline size, which might negatively affect blood clot formation in small sockets in certain cases^15,56^. This should also be considered when managing bleeding from extraction sockets, aiming to use the most suitable method of hemostasis.

Certain limitations in this study should be kept in mind. The low number of trials with patients on antiplatelet drugs did not allow for conducting meta-analysis including studies with only these patients. The overall small number of studies also resulted in performing only one analysis with patients on OAT in general, with regards to the time to reach hemostasis. The extracted teeth were not specified in the analyzed investigations (e.g., molar versus anterior teeth). This may have affected the occurrence of bleeding events, because more difficult cases might have a higher risk of postoperative bleeding events. Additionally, most of the included studies showed a potential risk of bias. Along with other factors, this led to having a lower level of evidence and less confidence in the outcome of this network meta-analysis. Therefore, future studies with more direct comparisons between the available agents would be of high importance, to further explore and validate the conclusions drawn in this meta-analysis, and to assess whether its outcome could be applied to patients under antiplatelet therapy as well.

## Conclusions

Within the limitations of this study, it is concluded that the use of cyanoacrylate tissue adhesive and tranexamic acid gives favorable results in reducing postoperative bleeding events following dental extractions. Although chitosan dental dressing and collagen exhibited a faster time to reach hemostasis, they led to a higher occurrence of bleeding events and ranked last among all other hemostatic methods.

## Methods

This study was conducted following the Preferred Reporting Items for Systematic Reviews and Meta-analyses (PRISMA), with the extension for network meta-analysis^57^, and was registered in the PROSPERO database (registration number CRD42023408207).

The PICOS protocol was implemented to develop a suitable focused question and include potential investigations:

- Population (P): patients under OAT who are in need of dental extractions.
- Intervention (I): the use of hemostatic agents following tooth extraction.
- Comparator (C): the use of conventional methods of hemostasis (i.e., gauze/cotton pressure, sutures).
- Outcomes (O): time to reach hemostasis, and peri-/postoperative bleeding events.
- Study design (S): randomized clinical trials (RCTs).

As a result, the focused question of this study was: in antithrombotic patients receiving dental extractions (P), what is the efficacy of different hemostatic agents (I) in bleeding control (O), compared with the conventional measures of hemostasis (C), based on the results of randomized clinical trials (S)?

### Search strategy

Database search was carried out in 3 search engines: PubMed/Medline, Cochrane Central Register of Controlled Trials (CENTRAL), and Scopus. In addition, an extensive search process was done in the grey literature, aiming to find more eligible studies, as well as unpublished investigations if available.

A combination of free keywords and Medical Subject Heading search terms (i.e., MeSH) was inserted in databases during the search process (Supplementary Table 13).

### Study selection

Eligible investigations had to fulfill the PICOS criteria stated previously. Studies where hemostasis was achieved with the help of other methods (e.g., electrocautery), reports that do not address the focused question (e.g., different surgical procedures, irrelevant measurements), in addition to trials where the control group stopped or modified their drug regimen were excluded from this meta-analysis.

Screening of potential studies was performed independently by two reviewers (B.M and S.J). This process was done using the Rayyan website (Rayyan, Qatar Computing Research Institute, Qatar Foundation)^58^. Whenever any disagreement took place regarding the inclusion/exclusion of any study, it was resolved by discussion or consulting a third reviewer (A.P). Upon the completion of the database/grey literature search, all the references of the included studies were also screened, to find more eligible articles, if available. The date of ending the literature search was March 20, 2023.

### Data extraction

The information recorded from the eligible studies were the type of patient allocation (i.e., different patients for each group or split-mouth), patient condition (i.e., on anti-thrombotic therapy or anti-coagulant/platelet medication when specified), the type of dental extraction (simple/surgical), the hemostatic agent(s) used, the conventional method used to stop bleeding (if any), the number of patients/extractions from all groups, the time to achieve hemostasis, and postoperative bleeding events and their time of occurrence.

### Risk of bias

The revised Cochrane risk-of-bias tool for randomized trials (RoB 2) was used to assess the risk of bias in the eligible studies in this network meta-analysis^59^. This tool contains 5 domains (randomization process, deviations from the intended interventions, missing outcome data, measurement of the outcome, and selection of the reported result) to check and evaluate. The level of bias for the included studies was determined using the Excel tool of RoB2.

### Statistical analysis

A frequentist network meta-analysis (NMA) was performed using R software, version 4.2.2, utilizing “netmeta” and “dmetar” packages, implementing the random effect model. To avoid network disconnection, and because gauze/cotton pressure was used alone or with sutures, or depending on the case in the control groups of some studies^33,35^, the reference group in this meta-analysis was set as “conventional”, which included the application of gauze/cotton pressure with/without the use of sutures. Considering the slight variations in the definition of hemostasis in the included studies, NMA results regarding the continuous data of the time to reach hemostasis were obtained utilizing the standardized mean difference (SMD) and 95% confidence interval (CI), while the dichotomous data on bleeding events were analyzed implementing the odds ratio (OR) and 95% CI. Negative outcome measures for continuous data, as well as values lower than 1 for dichotomous data, indicated beneficial effects, i.e., a greater reduction in the time to reach hemostasis or bleeding events, respectively. In addition, negative ranges of confidence intervals for continuous data and ranges that did not include the value of 1 in the dichotomous data revealed statistical significance. A subgroup analysis was done on studies recruiting only patients undergoing simple (i.e., non-surgical) tooth extraction, as well as those taking anticoagulants. Heterogeneity was measured with I^2^-static, in which I^2^ values of 25%, 50%, and 75% represented low, moderate, and high heterogeneity, respectively^60^. Moreover, inconsistency between direct and indirect estimates was evaluated by the net-split function in the “netmeta” package of R software and measured by Cochran’s Q statistics for multivariate meta-analysis^61^.

P-scores were used to rank the treatments, where a higher value means better performance^62^. Network graphs and forest plots were generated to illustrate the relevant comparisons and treatment rankings, respectively.

### Quality of evidence

The certainty of evidence was evaluated following the GRADE system^63^, which ranks the level of evidence as “high”, “moderate”, “low”, or “very low”, assessing several points (risk of bias, imprecision, inconsistency, indirectness, and publication bias), with a modification of adding “coherence” to judge the difference between direct and indirect evidence (i.e., transitivity) obtained from the network analysis. The quality of evidence was evaluated for both the mixed and indirect evidence, to assess the overall level of evidence from this study^64^. The tables of the quality of evidence from all possible comparisons under each outcome were generated using the CINeMA software^65^.

## Supplementary Information

Supplementary Information.

## Data Availability

The data that support the findings of this study are available from the corresponding author upon reasonable request.
