# Supplementary material for: The effect of different hemostatic agents following dental extraction in patients under oral antithrombotic therapy: a network meta-analysis
Source: Sci Rep. 2023 Aug 2;13:12519. doi: 10.1038/s41598-023-39023-7 (PMC10397210; doi:10.1038/s41598-023-39023-7)
Supplement: Supplementary file 1 — Supplementary Information. [file 41598_2023_39023_MOESM1_ESM.pdf]

Reasons for exclusion	Study title
healthy patients only or mixed healthy/under OAT	1- TETIS study: evaluation of new topical hemostatic agent TT-173 in tooth extraction.<br>2- Hemostasis of oral surgery wounds with the HemCon Dental Dressing.<br>3- Cyanoacrylate tissue adhesive or silk suture for closure of surgical wound following removal of an impacted mandibular third molar: A randomized controlled study.<br>4- Effectiveness of tranexamic acid and hemocoagulase for bleeding management in dental extraction patients – A pilot study<br>5- A double blind study on the efficacy of local application of hemocoagulase solution in wound healing<br>6- Eficacia y seguridad de la aplicación de subgalato y subsalicilato de bismuto como agentes hemostáticos después de la extracción quirúrgica de terceros molaresEficacia y seguridad de la aplicación de subgalato y subsalicilato de bismuto como agentes hemostáticos después de la extracción quirúrgica de terceros molars<br>7- A comparison between 2 absorbable hemostatic agents: gelatin sponge (Spongostan®) and oxidized regenerated cellulose (Surgicel®)<br>8- Efficacy of gelatamp in controlling the postoperative sequelae following mandibular posterior teeth extraction - A split-mouth study<br>9- Evaluation of fibrin sealant as a wound closure agent in mandibular third molar surgery--a prospective, randomized controlled clinical trial.
irrelevant/no measurements	1- Efficacy of type-1 collagen cones in extraction sockets following surgical removal of semi-impacted mandibular third molars: a randomized controlled trial.<br>2- Minor complications after mandibular third molar surgery: type, incidence, and possible prevention.<br>3- Randomized controlled trial on the effectiveness of absorbable collagen sponge after extraction of impacted mandibular third molar: split-mouth design.<br>4- In vivo hemostatic efficacy of polyurethane foam compared to collagen and gelatin.<br>5- Efficacy of a topical gel containing chitosan, chlorhexidine, allantoin and dexpanthenol for pain and inflammation control after third molar surgery: A randomized and placebo-controlled clinical trial.<br>6- The effects of a topical gel containing chitosan, 0,2% chlorhexidine, allantoin and despanthenol on the wound healing process subsequent to impacted lower third molar extraction.

	7- How effective is collagen resorbable membrane placement after partially impacted mandibular third molar surgery on postoperative morbidity? A prospective randomized comparative study.<br>8- Efficacy of chlorhexidine, dexpanthenol, allantoin and chitosan gel in comparison with bicarbonate oral rinse in controlling post-interventional inflammation, pain and cicatrization in subjects undergoing dental surgery.<br>9- Hemostasis control in dental extractions in patients receiving oral anticoagulant therapy: an approach with calcium sulfate.<br>10- Efficacy of gelatamp in controlling the postoperative sequelae following mandibular posterior teeth extraction - A split-mouth study.<br>11- Healing of Oral Surgical Wounds Using 3/0 Silk Suture and N-butyl Cyanoacrylate Tissue Adhesive<br>12- Efficacy of Haemocoagulase as a Topical Haemostatic Agent after Minor Oral Surgical Procedures—A Prospective Study<br>13- Effectiveness of haemocoagulase as local haemostatic agent after orthodontic extractions
several agents in one socket	1- Tranexamic acid mouthwash versus autologous fibrin glue in patients taking warfarin undergoing dental extractions: a randomized prospective clinical study.
study protocol	1- Tranexamic acid to reduce bleeding after dental extraction in patients treated with non-vitamin K oral anticoagulants: design and rationale of the EXTRACT-NOAC trial.
not RCT	1- Tissue adhesive and suturing for closure of the surgical wound after removal of impacted mandibular third molars: a comparative study.<br>2- [Hemostatic wound management in marcumar patients. Collagen fleece vs. tranexamic acid].<br>3- Tooth extraction without discontinuation of oral antithrombotic treatment: A prospective study<br>4- Use of thrombin powder after tooth extraction in patients receiving anticoagulant therapy<br>5- Application of the TachoComb to the patients receiving oral anticoagulants after tooth extraction<br>6- Use of tranexamic acid mouthwash to prevent postoperative bleeding in oral surgery patients on oral anticoagulant medication<br>7- Effectiveness of a hemostatic collagen dressing compared with regenerated oxidized cellulose in oral surgery<br>8- Effectiveness of hemcon dental dressing versus conventional method of haemostasis in 40 patients on oral antiplatelet drugs<br>9- EVALUATION OF COLLOIDAL SILVER GELATIN SPONGE (GELATAMP) IN PATIENTS RECEIVING

	ANTICOAGULANT AFTER TOOTH EXTRACTION (CLINICAL STUDY)<br>10- EFFICACY OF CHITOSAN AND ABSORBABLE GELATIN SPONGE ON HEMOSTASIS AND WOUND HEALING FOLLOWING TOOTH EXTRACTION "A COMPARATIVE STUDY"<br>11- EFFICACY OF PLATELET RICH FIBRIN VERSUS GELATIN SPONGE OR TRANEXAMIC ACID ON HEMOSTASIS AND WOUND HEALING FOLLOWING TOOTH EXTRACTION IN PATIENTS ON ANTICOAGULANT THERAPY
several/other surgical procedures	1- Hemostatic effect of tranexamic acid mouthwash in anticoagulant-treated patients undergoing oral surgery.<br>2- Prevention of postsurgical bleeding in oral surgery using tranexamic acid without dose modification of oral anticoagulants.<br>3- Use of N-butyl-2-cyanoacrylate in oral surgery: biological and clinical evaluation.<br>4- Calcium sulphate for control of bleeding after oral surgery in anticoagulant therapy patients<br>5- Successful management of bleeding after dental procedures with application of blood stopper: A single center prospective trial<br>6- Mouthwash with tranexamic acid in patients under oral anticoagulant therapy during dental surgery<br>7- Local hemostasis in the maxillofacial region with aid of fibrin adhesive system<br>8- Textured collagen, a hemostatic agent
hemostatic agent was used as supplement in some/all cases without specifying	1- Evaluation of dental extractions, suturing and INR on postoperative bleeding of patients maintained on oral anticoagulant therapy.<br>2- Oral surgery in patients on oral anticoagulant therapy: a randomized comparison of different intensity targets.<br>3- Haemostasis in oral surgical procedures involving patients with a ventricular assist device.<br>4- Dental extractions and risk of bleeding in patients taking single and dual antiplatelet treatment.<br>5- Managing anticoagulant patients undergoing dental extraction by using hemostatic agent: Tranexamic acid mouthrinse<br>6- Safety of dental extraction among consecutive patients on oral anticoagulant treatment managed using a specific dental management protocol.<br>7- Efficacy of fibrin sealant in patients on various levels of oral anticoagulant undergoing oral surgery

	8- Dental extractions in patients maintained on oral anticoagulant therapy: Comparison of INR value with occurrence of postoperative bleeding<br>9- Dental extractions in patients with bleeding disorders. The use of fibrin glue<br>10- Hemostatic Management of Tooth Extractions in Patients on Oral Antithrombotic Therapy
control group stopped their OAT	1- Tranexamic acid as a mouthwash in anticoagulant-treated patients undergoing oral surgery. An alternative method to discontinuing anticoagulant therapy.<br>2- Randomized, prospective trial comparing bridging therapy using low-molecular-weight heparin with maintenance of oral anticoagulation during extraction of teeth.<br>3- Local Delivery of the Hemostatic Agent Tranexamic Acid in Chronically Anticoagulated Patients
hemostatic agent was administered IV	1- Epsilon-Aminocaproic acid therapy for dental extractions in haemophilia and Christmas disease: a double blind controlled trial.
no hemostatic agent was used	1- Is it necessary to alter anticoagulation therapy for tooth extraction in patients taking direct oral anticoagulants?
no control group	1- The use of fibrin glue as an operative sealant in dental extraction in bleeding disorder patients

Supplementary Table 1. Articles qualified for full-text assessment and reasons for exclusion.

Unique ID	Study ID	D1	D2	D3	D4	D5	Overall
1	da Silva 2018	+	+	+	+	+	+
2	Brancaccio 2021	+	+	!	+	+	!
3	Cakarer 2013	!	+	+	+	!	!
4	Bajkin 2014	!	+	+	+	+	!
5	Giudice 2019	+	+	+	+	+	+
6	Soares 2015	!	+	!	+	+	!
7	Queiroz 2018	+	+	+	+	+	+
8	Kumar 2016	!	!	+	+	+	!
9	Rai 2019	!	+	+	+	+	!
10	Ockerman 2021	+	+	+	+	+	+
11	Pippi 2017	+	+	+	+	+	+
12	Radhakrishna 2023	!	+	+	!	+	!
13	Al-Belasy 2003	!	+	+	+	+	!
14	Halfpenny 2001	+	!	+	+	+	!
15	Pippi 2015	+	+	+	+	+	+
16	Sharma 2017	!	+	+	+	+	!
17	Muralidharan 2017	!	+	+	!	+	!
18	Puia 2020	+	+	+	+	+	+
19	Ragab 2019	!	+	+	!	+	!
20	Redwan 2020	+	+	+	+	+	+
21	Seethamsetty 2019	!	+	+	!	+	!
22	Ripollés-de Ramón 2014	!	+	+	-	!	-
23	Souto 1996	!	+	+	!	+	!

+ Low risk
! Some concerns
- High risk

D1 Randomisation process
D2 Deviations from the intended interventions
D3 Missing outcome data
D4 Measurement of the outcome
D5 Selection of the reported result

Supplementary Figure 1. Risk of bias in each domain of the included studies

**hemostatic agent**	**SMD [95% CI]**	**p-value**	**P-score***
Ankaferd blood stopper	-2.3900<br>[-8.7775; 3.9975]	0.4633	0.3213
Chitosan dental dressing	-9.7785<br>[-12.7812; -6.7758]	< 0.0001	0.8481
Collagen plug	-10.1285<br>[-15.5295; -4.7275]	0.0002	0.8705
Tranexamic acid	-2.8300<br>[-9.2175; 3.5575]	0.3852	0.3540
Conventional	Reference		0.1061
**Heterogeneity/Inconsistency**	$\tau^2 = 10.3906$; $\tau = 3.2235$; $I^2 = 98.3\%$		
**Q statistics****	Q= 288.97; p-value < 0.0001		

Supplementary Table 2. Results of the network meta-analysis for the time to reach hemostasis.

**Higher value of P-scores indicates better hemostatic efficacy*

***Q statistics to assess homogeneity / consistency*

hemostatic agent	OR [95% CI]	p-value	P-score
Cyanoacrylate tissue adhesive	0.0284 [0.0008; 1.0195]	0.0513	0.9248
Tranexamic acid	0.2676 [0.1032; 0.6940]	0.0067	0.7378
Ankaferd blood stopper	0.1901 [0.0080; 4.5276]	0.3048	0.7078
Feracrylum	0.3478 [0.0316; 3.8299]	0.3882	0.6184
Gelatin sponge	0.4724 [0.0781; 2.8582]	0.4142	0.5610
Bismuth subgallate	0.4585 [0.0115; 18.3266]	0.6786	0.5509
Oxidized regenerated cellulose	0.5536 [0.2471; 1.2405]	0.1509	0.5362
Epsilon aminocaproic acid (EACA)	0.8134 [0.2998; 2.2070]	0.6851	0.4023
Fibrin	1.1117 [0.2080; 5.9426]	0.9014	0.3301
Conventional	Reference		0.3170
Chitosan dental dressing	1.5556 [0.1305; 18.5483]	0.7268	0.2820
Collagen plug	12.0086 [0.7644; 188.6601]	0.0769	0.0318
**Heterogeneity/Inconsistency**	τ^2 = 0.1198; τ = 0.3461; I^2 = 12.5%		
**Q statistics**	Q= 3.10; p-value= 0.6851		

Supplementary Table 3. Results of the network meta-analysis for bleeding events in antithrombotic patients.

**hemostatic agent**	**OR [95% CI]**	**p-value**	**P-score**
Tranexamic acid	0.2406 [0.0754; 0.7674]	0.0161	0.7923
Cyanoacrylate tissue adhesive	0.1249 [0.0016; 9.4789]	0.3463	0.7888
Feracrylum	0.3589 [0.0257; 5.0107]	0.4461	0.6691
Bismuth subgallate	0.4106 [0.0060; 28.0867]	0.6797	0.6340
Oxidized regenerated cellulose	0.4808 [0.0120; 19.2651]	0.6973	0.6132
Epsilon aminocaproic acid (EACA)	0.7574 [0.1916; 2.9945]	0.6920	0.5234
Fibrin	1.0179 [0.0934; 11.0982]	0.9884	0.4627
Conventional	Reference		0.4478
Gelatin sponge	2.0751 [0.1267; 33.9801]	0.6088	0.3463
Collagen plug	10.8847 [0.3488; 339.6837]	0.1738	0.1234
Chitosan dental dressing	22.8740 [0.3183; 1643.8174]	0.1513	0.0992
**Heterogeneity/Inconsistency**	τ^2 = 0.4722; τ = 0.6872; I^2 = 32.3%		
**Q statistics**	Q= 1.33; p-value= 0.7217		

Supplementary Table 4. Results of the network meta-analysis for bleeding events in anticoagulated patients.

**hemostatic agent**	**OR [95% CI]**	**p-value**	**P-score**
Tranexamic acid	0.1180 [0.0196; 0.7116]	0.0198	0.8547
Ankaferd blood stopper	0.1901 [0.0065; 5.5552]	0.3350	0.7057
Feracrylum	0.3126 [0.0221; 4.4123]	0.3893	0.6346
Bismuth subgallate	0.2876 [0.0040; 20.7276]	0.5680	0.6301
Oxidized regenerated cellulose	0.5582 [0.1717; 1.8145]	0.3324	0.5261
Epsilon aminocaproic acid (EACA)	0.8958 [0.1799; 4.4606]	0.8932	0.3922
Fibrin	0.7127 [0.0595; 8.5405]	0.7893	0.4584
Conventional	Reference		0.3363
Gelatin sponge	0.4510 [0.0643; 3.1646]	0.4231	0.5744
Collagen plug	7.6227 [0.2291; 253.6151]	0.2560	0.0869
Chitosan dental dressing	1.4867 [0.1061; 20.8229]	0.7684	0.3006
**Heterogeneity/Inconsistency**	τ^2 = 0.4683; τ = 0.6843; I^2 = 34.3%		
**Q statistics**	Q= 6.13; p-value= 0.11		

Supplementary Table 5. Results of the network meta-analysis for bleeding events following simple (i.e., non-surgical) tooth extraction in antithrombotic patients.

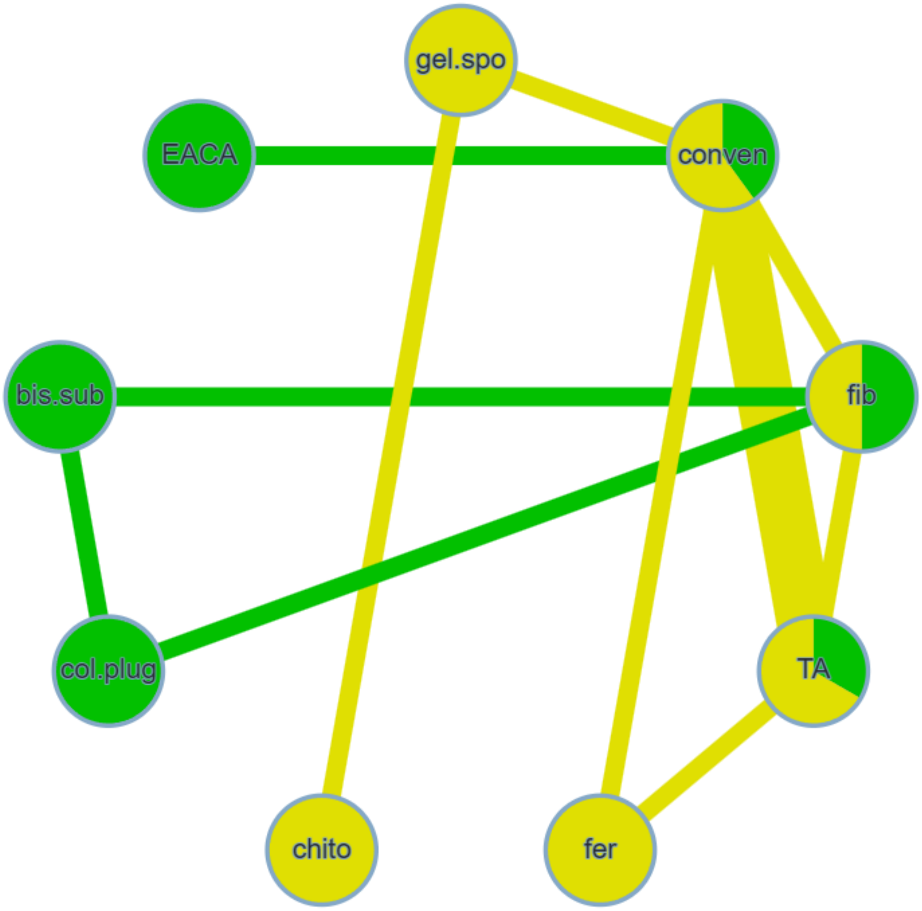

Supplementary Figure 2. Network meta-analysis geometry of the comparisons available on bleeding events after simple (i.e., non-surgical) tooth extraction in anticoagulated patients.

hemostatic agent	OR [95% CI]	p-value	P-score
Tranexamic acid	0.1169 [0.0106; 1.2890]	0.0797	0.8418
Feracrylum	0.3200 [0.0080; 12.7904]	0.5449	0.6606
Bismuth subgallate	0.2738 [0.0009; 79.8469]	0.6546	0.6739
Epsilon aminocaproic acid (EACA)	0.8958 [0.0369; 21.7212]	0.9461	0.5076
Fibrin	0.7096 [0.0207; 24.3195]	0.8491	0.5468
Conventional	Reference		0.4767
Gelatin sponge	2.0751 [0.0411; 104.8946]	0.7153	0.4040
Collagen plug	7.3869 [0.0442; 1233.7314]	0.4438	0.2269
Chitosan dental dressing	22.8740 [0.0706; 7413.2035]	0.2886	0.1617
**Heterogeneity/Inconsistency**	τ^2 = 2.4437; τ = 1.5632; I^2 = 54.3%		
**Q statistics**	Q= 4.37; p-value= 0.1127		

Supplementary Table 6. Results of the network meta-analysis for bleeding events following simple (i.e., non-surgical) tooth extraction in anticoagulated patients.

Bismuth subgallate	.	0.04 (0.00; 2.75)	.	.	.	0.33 (0.00; 27.96)	.	.
0.01 (0.00; 39.49)	Chitosan dental dressing	.	.	.	.	.	11.02 (0.16; 769.89)	.
0.04 (0.00; 2.45)	3.10 (0.00; 6983.86)	Collagen plug	.	.	.	11.25 (0.28; 455.70)	.	.
0.27 (0.00; 79.85)	22.87 (0.07; 7413.20)	7.39 (0.04; 1233.73)	Conventional methods	1.12 (0.05; 27.07)	3.32 (0.07; 156.12)	0.48 (0.01; 24.36)	0.48 (0.01; 24.36)	8.38 (0.76; 92.32)
0.31 (0.00; 205.27)	25.53 (0.03; 18805.82)	8.25 (0.02; 3427.68)	1.12 (0.05; 27.07)	Epsilon aminocaproic acid	.	.	.	.
0.86 (0.00; 645.88)	71.47 (0.08; 67951.11)	23.08 (0.05; 10883.80)	3.12 (0.08; 124.88)	2.80 (0.02; 366.65)	Feracrylum	.	.	3.10 (0.03; 290.31)
0.39 (0.00; 32.73)	32.23 (0.04; 28248.84)	10.41 (0.26; 421.81)	1.41 (0.04; 48.29)	1.26 (0.01 147.36)	0.45 (0.00; 61.70)	Fibrin	.	2.08 (0.04; 104.89)
0.13 (0.00; 130.84)	11.02 (0.16; 769.89)	3.56 (0.01; 2249.15)	0.48 (0.01; 24.36)	0.43 (0.00; 67.71)	0.15 (0.00; 33.62)	0.34 (0.00; 67.17)	Gelatin sponge	.
2.34 (0.01; 682.76)	195.59 (0.37; 102272.12)	63.16 (0.38; 10549.42)	8.55 (0.78; 94.25)	7.66 (0.14; 414.30)	2.74 (0.05; 141.38)	6.07 (0.18; 207.95)	17.74 (0.18; 1763.12)	Tranexamic acid

Supplementary Table 7. league table of the network meta-analysis of bleeding events after simple (i.e., non-surgical) tooth extractions in anticoagulated patients.

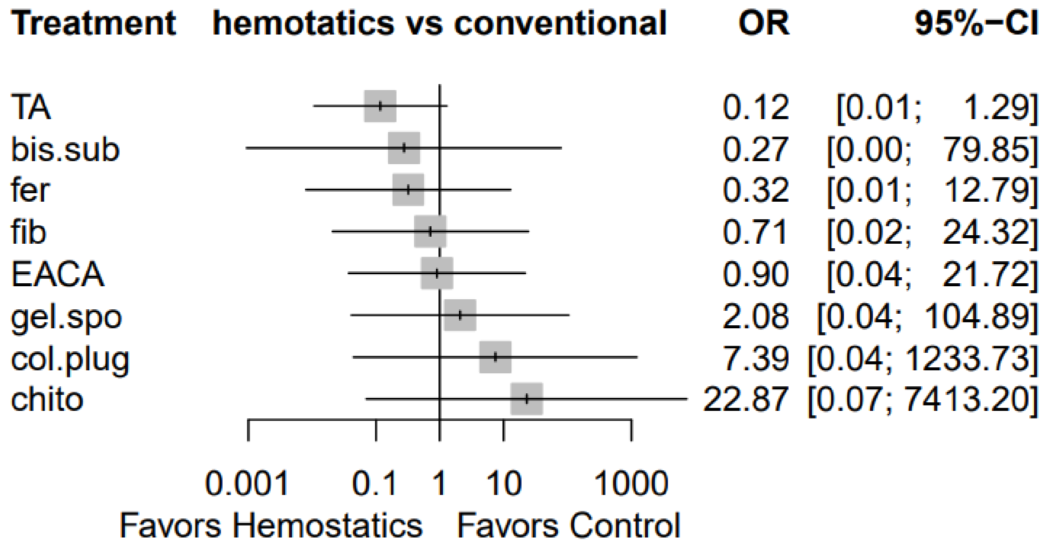

Supplementary Figure 3. Forest plot to show the ranking of the available interventions with regards to bleeding events after simple tooth extraction in anticoagulated patients.

Comparison	Number of studies	Within-study bias	Reporting bias	Indirectness	Imprecision	Heterogeneity	Incoherence	Confidence rating	Reason(s) for downgrading
conven:TA	1	No concerns	Low risk	No concerns	No concerns	Major concerns	Major concerns	Low	["Heterogeneity”, Incoherence"]
ankaferd:conven	1	Some concerns	Low risk	No concerns	No concerns	Major concerns	Major concerns	Very low	["Within-study bias”, “Heterogeneity", "Incoherence"]
chito:col.plug	2	No concerns	Low risk	No concerns	Major concerns	No concerns	Major concerns	Low	["Imprecision”, Incoherence"]
chito:conven	5	Some concerns	Low risk	No concerns	No concerns	Major concerns	Major concerns	Very low	["Within-study bias”, “Heterogeneity", "Incoherence"]
**Indirect evidence**									
ankaferd:TA	0	Some concerns	Low risk	No concerns	Major concerns	No concerns	Major concerns	Very low	["Within-study bias", "Imprecision”, Incoherence"]
chito:TA	0	No concerns	Low risk	No concerns	Major concerns	No concerns	Major concerns	Low	["Imprecision”, Incoherence"]
col.plug:TA	0	No concerns	Low risk	No concerns	Major concerns	No concerns	Major concerns	Low	["Imprecision”, Incoherence"]
ankaferd:chito	0	Some concerns	Low risk	No concerns	Major concerns	No concerns	Major concerns	Very low	["Within-study bias”, “Imprecision", "Incoherence"]
ankaferd:col.plug	0	Some concerns	Low risk	No concerns	No concerns	Major concerns	Major concerns	Very low	["Within-study bias", "Heterogeneity”, Incoherence"]
col.plug:conven	0	No concerns	Low risk	No concerns	No concerns	Major concerns	Major concerns	Low	["Heterogeneity”, Incoherence"]

Supplementary Table 8. Certainty of evidence in the network meta-analysis for the time to reach hemostasis.

Comparison	Number of studies	Within-study bias	Reporting bias	Indirectness	Imprecision	Heterogeneity	Incoherence	Confidence rating	Reason(s) for downgrading
EACA:TA	1	No concerns	Low risk	No concerns	Major concerns	No concerns	No concerns	Moderate	["Imprecision"]
conven:EACA	1	No concerns	Low risk	No concerns	Major concerns	No concerns	No concerns	Moderate	["Imprecision"]
conven:TA	4	No concerns	Low risk	No concerns	No concerns	Major concerns	No concerns	Moderate	["Heterogeneity"]
fer:TA	1	Some concerns	Low risk	No concerns	Major concerns	No concerns	No concerns	Low	["Within-study bias","Imprecision"]
fib:TA	1	Some concerns	Low risk	No concerns	Major concerns	No concerns	No concerns	Low	["Within-study bias","Imprecision"]
ankaferd:conven	1	Some concerns	Low risk	No concerns	Major concerns	No concerns	No concerns	Low	["Within-study bias","Imprecision"]
bis.sub:col.plug	1	No concerns	Low risk	No concerns	No concerns	Major concerns	No concerns	Moderate	["Heterogeneity"]
bis.sub:fib	1	No concerns	Low risk	No concerns	Major concerns	No concerns	No concerns	Moderate	["Imprecision"]
cellulose:conven	2	Some concerns	Low risk	No concerns	Major concerns	No concerns	No concerns	Low	["Within-study bias","Imprecision"]
cellulose:fib	1	Some concerns	Low risk	No concerns	Major concerns	No concerns	No concerns	Low	["Within-study bias","Imprecision"]
chito:conven	1	Some concerns	Low risk	No concerns	Major concerns	No concerns	No concerns	Low	["Within-study bias","Imprecision"]
chito:gel.spo	1	Some concerns	Low risk	No concerns	Major concerns	No concerns	No concerns	Low	["Within-study bias","Imprecision"]
col.plug:fib	1	No concerns	Low risk	No concerns	No concerns	Major concerns	No concerns	Moderate	["Heterogeneity"]
conven:fer	1	Some concerns	Low risk	No concerns	Major concerns	No concerns	No concerns	Low	["Within-study bias","Imprecision"]
conven:fib	1	Some concerns	Low risk	No concerns	Major concerns	No concerns	No concerns	Low	["Within-study bias","Imprecision"]
conven:gel.spo	2	Some concerns	Low risk	No concerns	Major concerns	No concerns	No concerns	Low	["Within-study bias","Imprecision"]
cyano:gel.spo	1	Some concerns	Low risk	No concerns	Major concerns	No concerns	No concerns	Low	["Within-study bias","Imprecision"]
**Indirect evidence**									
ankaferd:EACA	0	Some concerns	Low risk	No concerns	Major concerns	No concerns	No concerns	Low	["Within-study bias","Imprecision"]
bis.sub:EACA	0	No concerns	Low risk	No concerns	Major concerns	No concerns	No concerns	Low	["Imprecision"]
cellulose:EACA	0	No concerns	Low risk	No concerns	Major concerns	No concerns	No concerns	Moderate	["Imprecision"]
chito:EACA	0	Some concerns	Low risk	No concerns	Major concerns	No concerns	No concerns	Low	["Within-study bias","Imprecision"]

col.plug:EACA	0	No concerns	Low risk	No concerns	Major concerns	No concerns	No concerns	Moderate	["Imprecision"]
cyano:EACA	0	Some concerns	Low risk	No concerns	Major concerns	No concerns	No concerns	Low	["Within-study bias","Imprecision"]
EACA:fer	0	Some concerns	Low risk	No concerns	Major concerns	No concerns	No concerns	Low	["Within-study bias","Imprecision"]
EACA:fib	0	Some concerns	Low risk	No concerns	Major concerns	No concerns	No concerns	Low	["Within-study bias","Imprecision"]
EACA:gel.spo	0	Some concerns	Low risk	No concerns	Major concerns	No concerns	No concerns	Low	["Within-study bias","Imprecision"]
ankaferd:TA	0	Some concerns	Low risk	No concerns	Major concerns	No concerns	No concerns	Low	["Within-study bias","Imprecision"]
bis.sub:TA	0	No concerns	Low risk	No concerns	Major concerns	No concerns	No concerns	Moderate	["Imprecision"]
cellulose:TA	0	Some concerns	Low risk	No concerns	Major concerns	No concerns	No concerns	Low	["Within-study bias","Imprecision"]
chito:TA	0	Some concerns	Low risk	No concerns	Major concerns	No concerns	No concerns	Low	["Within-study bias","Imprecision"]
col.plug:TA	0	No concerns	Low risk	No concerns	No concerns	No concerns	No concerns	High	[]
cyano:TA	0	Some concerns	Low risk	No concerns	Major concerns	No concerns	No concerns	Low	["Within-study bias","Imprecision"]
gel.spo:TA	0	Some concerns	Low risk	No concerns	Major concerns	No concerns	No concerns	Low	["Within-study bias","Imprecision"]
ankaferd:bis.sub	0	Some concerns	Low risk	No concerns	Major concerns	No concerns	No concerns	Low	["Within-study bias","Imprecision"]
ankaferd:cellulose	0	Some concerns	Low risk	No concerns	Major concerns	No concerns	No concerns	Low	["Within-study bias","Imprecision"]
ankaferd:chito	0	Some concerns	Low risk	No concerns	Major concerns	No concerns	No concerns	Low	["Within-study bias","Imprecision"]
ankaferd:col.plug	0	Some concerns	Low risk	No concerns	Major concerns	No concerns	No concerns	Low	["Within-study bias","Imprecision"]
ankaferd:cyano	0	Some concerns	Low risk	No concerns	Major concerns	No concerns	No concerns	Low	["Within-study bias","Imprecision"]
ankaferd:fer	0	Some concerns	Low risk	No concerns	Major concerns	No concerns	No concerns	Low	["Within-study bias","Imprecision"]
ankaferd:fib	0	Some concerns	Low risk	No concerns	Major concerns	No concerns	No concerns	Low	["Within-study bias","Imprecision"]
ankaferd:gel.spo	0	Some concerns	Low risk	No concerns	Major concerns	No concerns	No concerns	Low	["Within-study bias","Imprecision"]
bis.sub:cellulose	0	No concerns	Low risk	No concerns	Major concerns	No concerns	No concerns	Moderate	["Imprecision"]
bis.sub:chito	0	Some concerns	Low risk	No concerns	Major concerns	No concerns	No concerns	Low	["Within-study bias","Imprecision"]
bis.sub:conven	0	No concerns	Low risk	No concerns	Major concerns	No concerns	No concerns	Moderate	["Imprecision"]

bis.sub:cyano	0	Some concerns	Low risk	No concerns	Major concerns	No concerns	No concerns	Low	["Within-study bias","Imprecision"]
bis.sub:fer	0	Some concerns	Low risk	No concerns	Major concerns	No concerns	No concerns	Low	["Within-study bias","Imprecision"]
bis.sub:gel.spo	0	Some concerns	Low risk	No concerns	Major concerns	No concerns	No concerns	Low	["Within-study bias","Imprecision"]
cellulose:chito	0	Some concerns	Low risk	No concerns	Major concerns	No concerns	No concerns	Low	["Within-study bias","Imprecision"]
cellulose:col.plug	0	Some concerns	Low risk	No concerns	No concerns	Major concerns	No concerns	Low	["Within-study bias","Heterogeneity"]
cellulose:cyano	0	Some concerns	Low risk	No concerns	Major concerns	No concerns	No concerns	Low	["Within-study bias","Imprecision"]
cellulose:fer	0	Some concerns	Low risk	No concerns	Major concerns	No concerns	No concerns	Low	["Within-study bias","Imprecision"]
cellulose:gel.spo	0	Some concerns	Low risk	No concerns	Major concerns	No concerns	No concerns	Low	["Within-study bias","Imprecision"]
chito:col.plug	0	Some concerns	Low risk	No concerns	Major concerns	No concerns	No concerns	Low	["Within-study bias","Imprecision"]
chito:cyano	0	Some concerns	Low risk	No concerns	No concerns	Major concerns	No concerns	Low	["Within-study bias","Heterogeneity"]
chito:fer	0	Some concerns	Low risk	No concerns	Major concerns	No concerns	No concerns	Low	["Within-study bias","Imprecision"]
chito:fib	0	Some concerns	Low risk	No concerns	Major concerns	No concerns	No concerns	Low	["Within-study bias","Imprecision"]
col.plug:conven	0	No concerns	Low risk	No concerns	Major concerns	No concerns	No concerns	Moderate	["Imprecision"]
col.plug:cyano	0	Some concerns	Low risk	No concerns	No concerns	No concerns	No concerns	Moderate	["Within-study bias"]
col.plug:fer	0	Some concerns	Low risk	No concerns	Major concerns	No concerns	No concerns	Low	["Within-study bias","Imprecision"]
col.plug:gel.spo	0	Some concerns	Low risk	No concerns	No concerns	Major concerns	No concerns	Low	["Within-study bias","Heterogeneity"]
conven:cyano	0	Some concerns	Low risk	No concerns	No concerns	Major concerns	No concerns	Low	["Within-study bias","Heterogeneity"]
cyano:fer	0	Some concerns	Low risk	No concerns	Major concerns	No concerns	No concerns	Low	["Within-study bias","Imprecision"]
cyano:fib	0	Some concerns	Low risk	No concerns	Major concerns	No concerns	No concerns	Low	["Within-study bias","Imprecision"]
fer:fib	0	Some concerns	Low risk	No concerns	Major concerns	No concerns	No concerns	Low	["Within-study bias","Imprecision"]
fer:gel.spo	0	Some concerns	Low risk	No concerns	Major concerns	No concerns	No concerns	Low	["Within-study bias","Imprecision"]
fib:gel.spo	0	Some concerns	Low risk	No concerns	Major concerns	No concerns	No concerns	Low	["Within-study bias","Imprecision"]

Supplementary Table 9. Certainty of evidence in the network meta-analysis for bleeding event in antithrombotic patients.

Comparison	Number of studies	Within-study bias	Reporting bias	Indirectness	Imprecision	Heterogeneity	Incoherence	Confidence rating	Reason(s) for downgrading
EACA:TA	1	No concerns	Low risk	No concerns	Major concerns	No concerns	No concerns	Moderate	["Imprecision"]
conven:EACA	1	No concerns	Low risk	No concerns	Major concerns	No concerns	No concerns	Moderate	["Imprecision"]
conven:TA	4	No concerns	Low risk	No concerns	No concerns	Major concerns	No concerns	Moderate	["Heterogeneity"]
fer:TA	1	Some concerns	Low risk	No concerns	Major concerns	No concerns	No concerns	Low	["Within-study bias","Imprecision"]
fib:TA	1	Some concerns	Low risk	No concerns	Major concerns	No concerns	No concerns	Low	["Within-study bias","Imprecision"]
bis.sub:col.plug	1	No concerns	Low risk	No concerns	No concerns	Major concerns	No concerns	Moderate	["Heterogeneity"]
bis.sub:fib	1	No concerns	Low risk	No concerns	Major concerns	No concerns	No concerns	Moderate	["Imprecision"]
cellulose:fib	1	Some concerns	Low risk	No concerns	Major concerns	No concerns	No concerns	Low	["Within-study bias","Imprecision"]
chito:gel.spo	1	Some concerns	Low risk	No concerns	Major concerns	No concerns	No concerns	Low	["Within-study bias","Imprecision"]
col.plug:fib	1	No concerns	Low risk	No concerns	No concerns	Major concerns	No concerns	Moderate	["Heterogeneity"]
conven:fer	1	Some concerns	Low risk	No concerns	Major concerns	No concerns	No concerns	Low	["Within-study bias","Imprecision"]
conven:fib	1	Some concerns	Low risk	No concerns	Major concerns	No concerns	No concerns	Low	["Within-study bias","Imprecision"]
conven:gel.spo	1	Some concerns	Low risk	No concerns	Major concerns	No concerns	No concerns	Low	["Within-study bias","Imprecision"]
cyano:gel.spo	1	Some concerns	Low risk	No concerns	Major concerns	No concerns	No concerns	Low	["Within-study bias","Imprecision"]
**Indirect evidence**									
bis.sub:EACA	0	No concerns	Low risk	No concerns	Major concerns	No concerns	No concerns	Moderate	["Imprecision"]
cellulose:EACA	0	Some concerns	Low risk	No concerns	Major concerns	No concerns	No concerns	Low	["Within-study bias","Imprecision"]
chito:EACA	0	Some concerns	Low risk	No concerns	Major concerns	No concerns	No concerns	Low	["Within-study bias","Imprecision"]
col.plug:EACA	0	No concerns	Low risk	No concerns	Major concerns	No concerns	No concerns	Moderate	["Imprecision"]
cyano:EACA	0	Some concerns	Low risk	No concerns	Major concerns	No concerns	No concerns	Low	["Within-study bias","Imprecision"]
EACA:fer	0	Some concerns	Low risk	No concerns	Major concerns	No concerns	No concerns	Low	["Within-study bias","Imprecision"]
EACA:fib	0	Some concerns	Low risk	No concerns	Major concerns	No concerns	No concerns	Low	["Within-study bias","Imprecision"]

EACA:gel.spo	0	Some concerns	Low risk	No concerns	Major concerns	No concerns	No concerns	Low	["Within-study bias","Imprecision"]
bis.sub:TA	0	No concerns	Low risk	No concerns	Major concerns	No concerns	No concerns	Moderate	["Imprecision"]
cellulose:TA	0	Some concerns	Low risk	No concerns	Major concerns	No concerns	No concerns	Low	["Within-study bias","Imprecision"]
chito:TA	0	Some concerns	Low risk	No concerns	No concerns	Major concerns	No concerns	Low	["Within-study bias","Heterogeneity"]
col.plug:TA	0	No concerns	Low risk	No concerns	No concerns	Major concerns	No concerns	Moderate	["Heterogeneity"]
cyano:TA	0	Some concerns	Low risk	No concerns	Major concerns	No concerns	No concerns	Low	["Within-study bias","Imprecision"]
gel.spo:TA	0	Some concerns	Low risk	No concerns	Major concerns	No concerns	No concerns	Low	["Within-study bias","Imprecision"]
bis.sub:cellulose	0	No concerns	Low risk	No concerns	Major concerns	No concerns	No concerns	Moderate	["Imprecision"]
bis.sub:chito	0	Some concerns	Low risk	No concerns	Major concerns	No concerns	No concerns	Low	["Within-study bias","Imprecision"]
bis.sub:conven	0	No concerns	Low risk	No concerns	Major concerns	No concerns	No concerns	Moderate	["Imprecision"]
bis.sub:cyano	0	Some concerns	Low risk	No concerns	Major concerns	No concerns	No concerns	Low	["Within-study bias","Imprecision"]
bis.sub:fer	0	Some concerns	Low risk	No concerns	Major concerns	No concerns	No concerns	Low	["Within-study bias","Imprecision"]
bis.sub:gel.spo	0	Some concerns	Low risk	No concerns	Major concerns	No concerns	No concerns	Low	["Within-study bias","Imprecision"]
cellulose:chito	0	Some concerns	Low risk	No concerns	Major concerns	No concerns	No concerns	Low	["Within-study bias","Imprecision"]
cellulose:col.plug	0	No concerns	Low risk	No concerns	Major concerns	No concerns	No concerns	Moderate	["Imprecision"]
cellulose:conven	0	Some concerns	Low risk	No concerns	Major concerns	No concerns	No concerns	Low	["Within-study bias","Imprecision"]
cellulose:cyano	0	Some concerns	Low risk	No concerns	Major concerns	No concerns	No concerns	Low	["Within-study bias","Imprecision"]
cellulose:fer	0	Some concerns	Low risk	No concerns	Major concerns	No concerns	No concerns	Low	["Within-study bias","Imprecision"]
cellulose:gel.spo	0	Some concerns	Low risk	No concerns	Major concerns	No concerns	No concerns	Low	["Within-study bias","Imprecision"]
chito:col.plug	0	Some concerns	Low risk	No concerns	Major concerns	No concerns	No concerns	Low	["Within-study bias","Imprecision"]
chito:conven	0	Some concerns	Low risk	No concerns	Major concerns	No concerns	No concerns	Low	["Within-study bias","Imprecision"]
chito:cyano	0	Some concerns	Low risk	No concerns	No concerns	Major concerns	No concerns	Low	["Within-study bias","Heterogeneity"]
chito:fer	0	Some concerns	Low risk	No concerns	Major concerns	No concerns	No concerns	Low	["Within-study bias","Imprecision"]
chito:fib	0	Some concerns	Low risk	No concerns	Major concerns	No concerns	No concerns	Low	["Within-study bias","Imprecision"]
col.plug:conven	0	No concerns	Low risk	No concerns	Major concerns	No concerns	No concerns	Moderate	["Imprecision"]

col.plug:cyano	0	Some concerns	Low risk	No concerns	Major concerns	No concerns	No concerns	Low	["Within-study bias","Imprecision"]
col.plug:fer	0	Some concerns	Low risk	No concerns	Major concerns	No concerns	No concerns	Low	["Within-study bias","Imprecision"]
col.plug:gel.spo	0	Some concerns	Low risk	No concerns	Major concerns	No concerns	No concerns	Low	["Within-study bias","Imprecision"]
conven:cyano	0	Some concerns	Low risk	No concerns	Major concerns	No concerns	No concerns	Low	["Within-study bias","Imprecision"]
cyano:fer	0	Some concerns	Low risk	No concerns	Major concerns	No concerns	No concerns	Low	["Within-study bias","Imprecision"]
cyano:fib	0	Some concerns	Low risk	No concerns	Major concerns	No concerns	No concerns	Low	["Within-study bias","Imprecision"]
fer:fib	0	Some concerns	Low risk	No concerns	Major concerns	No concerns	No concerns	Low	["Within-study bias","Imprecision"]
fer:gel.spo	0	Some concerns	Low risk	No concerns	Major concerns	No concerns	No concerns	Low	["Within-study bias","Imprecision"]
fib:gel.spo	0	Some concerns	Low risk	No concerns	Major concerns	No concerns	No concerns	Low	["Within-study bias","Imprecision"]

Supplementary Table 10. Certainty of evidence in the network meta-analysis for bleeding event in anticoagulated patients.

Comparison	Number of studies	Within-study bias	Reporting bias	Indirectness	Imprecision	Heterogeneity	Incoherence	Confidence rating	Reason(s) for downgrading
conven:EACA	1	No concerns	Low risk	No concerns	Major concerns	No concerns	No concerns	Moderate	["Imprecision"]
conven:TA	3	Some concerns	Low risk	No concerns	No concerns	No concerns	No concerns	Moderate	["Within-study bias","Imprecision"]
fer:TA	1	Some concerns	Low risk	No concerns	Major concerns	No concerns	No concerns	Low	["Within-study bias","Imprecision"]
fib:TA	1	Some concerns	Low risk	No concerns	Major concerns	No concerns	No concerns	Low	["Within-study bias","Imprecision"]
ankaferd:conven	1	Some concerns	Low risk	No concerns	Major concerns	No concerns	No concerns	Low	["Within-study bias","Imprecision"]
bis.sub:col.plug	1	No concerns	Low risk	No concerns	No concerns	Major concerns	No concerns	Moderate	["Heterogeneity"]
bis.sub:fib	1	No concerns	Low risk	No concerns	Major concerns	No concerns	No concerns	Moderate	["Imprecision"]
cellulose:conven	2	Some concerns	Low risk	No concerns	Major concerns	No concerns	No concerns	Low	["Within-study bias","Imprecision"]
chito:conven	1	Some concerns	Low risk	No concerns	Major concerns	No concerns	No concerns	Low	["Within-study bias","Imprecision"]
chito:gel.spo	1	Some concerns	Low risk	No concerns	Major concerns	No concerns	No concerns	Low	["Within-study bias","Imprecision"]
col.plug:fib	1	No concerns	Low risk	No concerns	No concerns	Major concerns	No concerns	Moderate	["Heterogeneity"]
conven:fer	1	Some concerns	Low risk	No concerns	Major concerns	No concerns	No concerns	Low	["Within-study bias","Imprecision"]
conven:fib	1	Some concerns	Low risk	No concerns	Major concerns	No concerns	No concerns	Low	["Within-study bias","Imprecision"]
conven:gel.spo	2	Some concerns	Low risk	No concerns	Major concerns	No concerns	No concerns	Low	["Within-study bias","Imprecision"]
**Indirect evidence**									
EACA:TA	0	No concerns	Low risk	No concerns	Major concerns	No concerns	No concerns	Moderate	["Imprecision"]
ankaferd:EACA	0	Some concerns	Low risk	No concerns	Major concerns	No concerns	No concerns	Low	["Within-study bias","Imprecision"]
bis.sub:EACA	0	No concerns	Low risk	No concerns	Major concerns	No concerns	No concerns	Moderate	["Imprecision"]
cellulose:EACA	0	No concerns	Low risk	No concerns	Major concerns	No concerns	No concerns	Moderate	["Imprecision"]
chito:EACA	0	Some concerns	Low risk	No concerns	Major concerns	No concerns	No concerns	Low	["Within-study bias","Imprecision"]
col.plug:EACA	0	No concerns	Low risk	No concerns	Major concerns	No concerns	No concerns	Moderate	["Imprecision"]
EACA:fer	0	Some concerns	Low risk	No concerns	Major concerns	No concerns	No concerns	Low	["Within-study bias","Imprecision"]

EACA:fib	0	Some concerns	Low risk	No concerns	Major concerns	No concerns	No concerns	Low	["Within-study bias","Imprecision"]
EACA:gel.spo	0	Some concerns	Low risk	No concerns	Major concerns	No concerns	No concerns	Low	["Within-study bias","Imprecision"]
ankaferd:TA	0	Some concerns	Low risk	No concerns	Major concerns	No concerns	No concerns	Low	["Within-study bias","Imprecision"]
bis.sub:TA	0	No concerns	Low risk	No concerns	Major concerns	No concerns	No concerns	Moderate	["Imprecision"]
cellulose:TA	0	Some concerns	Low risk	No concerns	Major concerns	No concerns	No concerns	Low	["Within-study bias","Imprecision"]
chito:TA	0	Some concerns	Low risk	No concerns	Major concerns	No concerns	No concerns	Low	["Within-study bias","Imprecision"]
col.plug:TA	0	Some concerns	Low risk	No concerns	No concerns	Major concerns	No concerns	Low	["Within-study bias","Heterogeneity"]
gel.spo:TA	0	Some concerns	Low risk	No concerns	Major concerns	No concerns	No concerns	Low	["Within-study bias","Imprecision"]
ankaferd:bis.sub	0	Some concerns	Low risk	No concerns	Major concerns	No concerns	No concerns	Low	["Within-study bias","Imprecision"]
ankaferd:cellulose	0	Some concerns	Low risk	No concerns	Major concerns	No concerns	No concerns	Low	["Within-study bias","Imprecision"]
ankaferd:chito	0	Some concerns	Low risk	No concerns	Major concerns	No concerns	No concerns	Low	["Within-study bias","Imprecision"]
ankaferd:col.plug	0	Some concerns	Low risk	No concerns	Major concerns	No concerns	No concerns	Low	["Within-study bias","Imprecision"]
ankaferd:fer	0	Some concerns	Low risk	No concerns	Major concerns	No concerns	No concerns	Low	["Within-study bias","Imprecision"]
ankaferd:fib	0	Some concerns	Low risk	No concerns	Major concerns	No concerns	No concerns	Low	["Within-study bias","Imprecision"]
ankaferd:gel.spo	0	Some concerns	Low risk	No concerns	Major concerns	No concerns	No concerns	Low	["Within-study bias","Imprecision"]
bis.sub:cellulose	0	Some concerns	Low risk	No concerns	Major concerns	No concerns	No concerns	Low	["Within-study bias","Imprecision"]
bis.sub:chito	0	Some concerns	Low risk	No concerns	Major concerns	No concerns	No concerns	Low	["Within-study bias","Imprecision"]
bis.sub:conven	0	No concerns	Low risk	No concerns	Major concerns	No concerns	No concerns	Moderate	["Imprecision"]
bis.sub:fer	0	Some concerns	Low risk	No concerns	Major concerns	No concerns	No concerns	Low	["Within-study bias","Imprecision"]
bis.sub:gel.spo	0	Some concerns	Low risk	No concerns	Major concerns	No concerns	No concerns	Low	["Within-study bias","Imprecision"]
cellulose:chito	0	Some concerns	Low risk	No concerns	Major concerns	No concerns	No concerns	Low	["Within-study bias","Imprecision"]
cellulose:col.plug	0	Some concerns	Low risk	No concerns	Major concerns	No concerns	No concerns	Low	["Within-study bias","Imprecision"]
cellulose:fer	0	Some concerns	Low risk	No concerns	Major concerns	No concerns	No concerns	Low	["Within-study bias","Imprecision"]

cellulose:fib	0	Some concerns	Low risk	No concerns	Major concerns	No concerns	No concerns	Low	["Within-study bias","Imprecision"]
cellulose:gel.spo	0	Some concerns	Low risk	No concerns	Major concerns	No concerns	No concerns	Low	["Within-study bias","Imprecision"]
chito:col.plug	0	Some concerns	Low risk	No concerns	Major concerns	No concerns	No concerns	Low	["Within-study bias","Imprecision"]
chito:fer	0	Some concerns	Low risk	No concerns	Major concerns	No concerns	No concerns	Low	["Within-study bias","Imprecision"]
chito:fib	0	Some concerns	Low risk	No concerns	Major concerns	No concerns	No concerns	Low	["Within-study bias","Imprecision"]
col.plug:conven	0	Some concerns	Low risk	No concerns	Major concerns	No concerns	No concerns	Low	["Within-study bias","Imprecision"]
col.plug:fer	0	Some concerns	Low risk	No concerns	Major concerns	No concerns	No concerns	Low	["Within-study bias","Imprecision"]
col.plug:gel.spo	0	Some concerns	Low risk	No concerns	Major concerns	No concerns	No concerns	Low	["Within-study bias","Imprecision"]
fer:fib	0	Some concerns	Low risk	No concerns	Major concerns	No concerns	No concerns	Low	["Within-study bias","Imprecision"]
fer:gel.spo	0	Some concerns	Low risk	No concerns	Major concerns	No concerns	No concerns	Low	["Within-study bias","Imprecision"]
fib:gel.spo	0	Some concerns	Low risk	No concerns	Major concerns	No concerns	No concerns	Low	["Within-study bias","Imprecision"]

Supplementary Table 11. Certainty of evidence in the network meta-analysis for bleeding event after simple (i.e., non-surgical) tooth extractions in antithrombotic patients.

Comparison	Number of studies	Within-study bias	Reporting bias	Indirectness	Imprecision	Heterogeneity	Incoherence	Confidence rating	Reason(s) for downgrading
conven:EACA	1	No concerns	Low risk	No concerns	Major concerns	No concerns	No concerns	Moderate	["Imprecision"]
conven:TA	3	Some concerns	Low risk	No concerns	Major concerns	No concerns	No concerns	Low	["Within-study bias","Imprecision"]
fer:TA	1	Some concerns	Low risk	No concerns	Major concerns	No concerns	No concerns	Low	["Within-study bias","Imprecision"]
fib:TA	1	Some concerns	Low risk	No concerns	Major concerns	No concerns	No concerns	Low	["Within-study bias","Imprecision"]
bis.sub:col.plug	1	No concerns	Low risk	No concerns	No concerns	Major concerns	No concerns	Moderate	["Heterogeneity"]
bis.sub:fib	1	No concerns	Low risk	No concerns	Major concerns	No concerns	No concerns	Low	["Imprecision"]
chito:gel.spo	1	Some concerns	Low risk	No concerns	Major concerns	No concerns	No concerns	Low	["Within-study bias","Imprecision"]
col.plug:fib	1	No concerns	Low risk	No concerns	No concerns	Major concerns	No concerns	Moderate	["Heterogeneity"]
conven:fer	1	Some concerns	Low risk	No concerns	Major concerns	No concerns	No concerns	Low	["Within-study bias","Imprecision"]
conven:fib	1	Some concerns	Low risk	No concerns	Major concerns	No concerns	No concerns	Low	["Within-study bias","Imprecision"]
conven:gel.spo	1	Some concerns	Low risk	No concerns	Major concerns	No concerns	No concerns	Low	["Within-study bias","Imprecision"]
**Indirect evidence**									
EACA:TA	0	No concerns	Low risk	No concerns	Major concerns	No concerns	No concerns	Moderate	["Imprecision"]
bis.sub:EACA	0	No concerns	Low risk	No concerns	Major concerns	No concerns	No concerns	Moderate	["Imprecision"]
chito:EACA	0	Some concerns	Low risk	No concerns	Major concerns	No concerns	No concerns	Low	["Within-study bias","Imprecision"]
col.plug:EACA	0	No concerns	Low risk	No concerns	Major concerns	No concerns	No concerns	Moderate	["Imprecision"]
EACA:fer	0	Some concerns	Low risk	No concerns	Major concerns	No concerns	No concerns	Low	["Within-study bias","Imprecision"]
EACA:fib	0	Some concerns	Low risk	No concerns	Major concerns	No concerns	No concerns	Low	["Within-study bias","Imprecision"]
EACA:gel.spo	0	No concerns	Low risk	No concerns	Major concerns	No concerns	No concerns	Moderate	["Imprecision"]
bis.sub:TA	0	No concerns	Low risk	No concerns	Major concerns	No concerns	No concerns	Moderate	["Imprecision"]
chito:TA	0	Some concerns	Low risk	No concerns	No concerns	Major concerns	No concerns	Low	["Within-study bias","Heterogeneity"]
col.plug:TA	0	Some concerns	Low risk	No concerns	No concerns	Major concerns	No concerns	Low	["Within-study bias","Heterogeneity"]

gel.spo:TA	0	Some concerns	Low risk	No concerns	Major concerns	No concerns	No concerns	Low	["Within-study bias","Imprecision"]
bis.sub:chito	0	Some concerns	Low risk	No concerns	Major concerns	No concerns	No concerns	Low	["Within-study bias","Imprecision"]
bis.sub:conven	0	No concerns	Low risk	No concerns	Major concerns	No concerns	No concerns	Moderate	["Imprecision"]
bis.sub:fer	0	Some concerns	Low risk	No concerns	Major concerns	No concerns	No concerns	Low	["Within-study bias","Imprecision"]
bis.sub:gel.spo	0	Some concerns	Low risk	No concerns	Major concerns	No concerns	No concerns	Low	["Within-study bias","Imprecision"]
chito:col.plug	0	Some concerns	Low risk	No concerns	Major concerns	No concerns	No concerns	Low	["Within-study bias","Imprecision"]
chito:conven	0	Some concerns	Low risk	No concerns	Major concerns	No concerns	No concerns	Low	["Within-study bias","Imprecision"]
chito:fer	0	Some concerns	Low risk	No concerns	Major concerns	No concerns	No concerns	Low	["Within-study bias","Imprecision"]
chito:fib	0	Some concerns	Low risk	No concerns	Major concerns	No concerns	No concerns	Low	["Within-study bias","Imprecision"]
col.plug:conven	0	Some concerns	Low risk	No concerns	Major concerns	No concerns	No concerns	Low	["Within-study bias","Imprecision"]
col.plug:fer	0	Some concerns	Low risk	No concerns	Major concerns	No concerns	No concerns	Low	["Within-study bias","Imprecision"]
col.plug:gel.spo	0	Some concerns	Low risk	No concerns	Major concerns	No concerns	No concerns	Low	["Within-study bias","Imprecision"]
fer:fib	0	Some concerns	Low risk	No concerns	Major concerns	No concerns	No concerns	Low	["Within-study bias","Imprecision"]
fer:gel.spo	0	Some concerns	Low risk	No concerns	Major concerns	No concerns	No concerns	Low	["Within-study bias","Imprecision"]
fib:gel.spo	0	Some concerns	Low risk	No concerns	Major concerns	No concerns	No concerns	Low	["Within-study bias","Imprecision"]

Supplementary Table 12. Certainty of evidence in the network meta-analysis for bleeding event after simple (i.e., non-surgical) tooth extractions in anticoagulated patients.

Database	Keywords
PubMed	“coagulants OR hemostatics OR Hemostatic agent OR Tissue sealant OR fibrin sealant OR Antifibrinolytic Agents OR Hemocoagulase OR Chitosan OR hemcon OR tranexamic acid OR Cyanoacrylates OR Bone wax OR Gelfoam OR Surgicel OR collagen sponge OR gelatin sponge OR hemostatic Collagen OR collaplug OR topical thrombin AND dental extraction controlled trial OR third molar removal controlled trial”
Scopus	"hemostatic" AND "tooth extraction" AND "bleeding"<br>"hemostatic" AND "dental extraction" AND "bleeding"
Cochrane library	"hemostatic" AND "tooth extraction" AND "bleeding"<br>"hemostatic" AND "dental extraction" AND "bleeding"

Supplementary Table 13. Keywords used in the database search process.
